# Kaempferol targeting on the fibroblast growth factor receptor 3-ribosomal S6 kinase 2 signaling axis prevents the development of rheumatoid arthritis

**DOI:** 10.1038/s41419-018-0433-0

**Published:** 2018-03-14

**Authors:** Cheol-Jung Lee, Su-Jin Moon, Jeong-Hee Jeong, Sangbae Lee, Mee-Hyun Lee, Sun-Mi Yoo, Hye Suk Lee, Han Chang Kang, Joo Young Lee, Weon Sun Lee, Hee-Jin Lee, Eun-Kyung Kim, Joo-Yeon Jhun, Mi-La Cho, Jun-Ki Min, Yong-Yeon Cho

**Affiliations:** 1 Integrated Research Institute of Pharmaceutical Sciences & BK21 PLUS Team for Creative Leader Program for Pharmacomics-based Future Pharmacy, College of Pharmacy, The Catholic University of Korea, 43, Jibong-ro, Wonmi-gu, Bucheon-si, Gyeonggi-do, 420-743 Republic of Korea; 2 Department of Internal Medicine, College of Medicine, Division for Rheumatology, The Catholic University of Korea, 505, Banpo-dong, Seocho-gu, Seoul 137-701 Republic of Korea; 3 The Rheumatism Research Center, Catholic Research Institute of Medical Science, The Catholic University of Korea, 505, Banpo-dong, Seocho-gu, Seoul 137-701 Republic of Korea; 4 Division of Immunology, Beckman Research Institute of the City of Hope, 1500, E. Duarte Rd, Duarte, CA 91010 USA; 5 China-US(Henan) Hormel Cancer Institute, No. 127, Dongming Road, Jinshui District, Zhengzhou, 450008 Henan China; 6 Clinical Medicine Research Institute of Bucheon St. Mary’s Hospital, The Catholic University of Korea, 327, Sosa-ro, Wonmi-gu, Bucheon-si, Gyeonggi-do, 420-717 Republic of Korea

## Abstract

Rheumatoid arthritis (RA) is a systemic inflammatory disease that mainly affects the synovial joints. Although involvement of the fibroblast growth factor (FGF) signaling pathway has been suggested as an important modulator in RA development, no clear evidence has been provided. In this study, we found that synovial fluid basic FGF (bFGF) concentration was significantly higher in RA than in osteoarthritis (OA) patients. bFGF stimulates proliferation and migration of human fibroblast-like synoviocytes (FLSs) by activation of the bFGF-FGF receptor 3 (FGFR3)-ribosomal S6 kinase 2 (RSK2) signaling axis. Moreover, a molecular docking study revealed that kaempferol inhibited FGFR3 activity by binding to the active pocket of the FGFR3 kinase domain. Kaempferol forms hydrogen bonds with the FGFR3 backbone oxygen of Glu555 and Ala558 and the side chain of Lys508. Notably, the inhibition of bFGF-FGFR3–RSK2 signaling by kaempferol suppresses the proliferation and migration of RA FLSs and the release of activated T-cell-mediated inflammatory cytokines, such as IL-17, IL-21, and TNF-α. We further found that activated phospho-FGFR3 and -RSK2 were more highly observed in RA than in OA synovium. The hyperplastic lining and sublining lymphoid aggregate layers of RA synovium showed p-RSK2-expressing CD68^+^ macrophages with high frequency, while pRSK2-expressing CD4^+^ T-cells was observed at a lower frequency. Notably, kaempferol administration in collagen-induced arthritis mice relieved the frequency and severity of arthritis. Kaempferol reduced osteoclast differentiation in vitro and in vivo relative to the controls and was associated with the inhibition of osteoclast markers, such as tartrate-resistant acid phosphatase, integrin β3, and MMP9. Conclusively, our data suggest that bFGF-induced FGFR3–RSK2 signaling may play a critical role during the initiation and progression of RA in terms of FLS proliferation and enhanced osteoclastogenesis, and that kaempferol may be effective as a new treatment for RA.

## Introduction

Rheumatoid arthritis (RA) is a chronic inflammatory disease characterized by infiltration of immune cells into the synovium and hyperplasia of the synovial lining. Synovial lining cells in RA joints increase to 10–15 cell layers^1–3^ due to the influx and proliferation of inflammatory cells, which eventually manifest as pannus formation, which grows in a tumor-like fashion and is a pathognomic finding of RA^4^. Since the angiogenesis and proliferation of fibroblast-like synoviocytes (FLSs) play pivotal roles in mechanisms involved in RA pathogenesis^5^, altered activities of angiogenic and growth factors in RA synovium or synovial fluids (SF) have been considered as treatment targets for the disease^5–7^.

Fibroblast growth factor (FGF) is a family of heparin-binding growth factors that shows increased concentration in RA SF compared with that in osteoarthritis (OA)^6^. In a previous study, basic FGF (bFGF) concentration in RA SF better reflected the severity of joint destruction compared with other cytokines, such as tumor necrosis factor α (TNF-α), interleukin (IL)-1, or IL-6^6^. In addition, bFGF overexpression in experimental arthritis mice resulted in worsened arthritis severity, and it depended on enhanced angiogenesis and osteoclastogenesis. Previous studies have shown the anti-apoptotic effects of bFGF in RA FLSs^8^ and its RANKL-inducing properties on RA FLSs^9^, which are findings that predict the activation of osteoclasts and structural damage to the affected joints. In terms of angiogenesis, bFGF activity in endothelial cells stimulates angiogenic events partly by upregulating vascular endothelial growth factor^10^. However, the pathophysiological roles of bFGF in RA and its signaling in immune cells or FLSs have not been well understood.

Proinflammatory cytokines such as TNF-α, IL-1, and IL-6 induce inflammatory reaction and chemokine production in FLSs, resulting in the increased influx of additional proinflammatory cells, including macrophages, into the synovium^11^. It has become clear that these proinflammatory cytokines work together with other mediators, such as IL-17 in an additive or synergistic way^12^. Traditionally, the imbalance between type 1 helper T (Th1) and type 2 helper T (Th2) subsets has been suggested to lie at the center of RA pathogenesis^13^. However, in the past decade, the key paradigm has changed because numerous studies have identified the pivotal roles of IL-17 and IL-17-expressing CD4^+^ T-cells, known as Th17 cells, in RA development and progression^14^. Prostaglandin E2 also plays a key role in FLS activation induced by proinflammatory cytokines and epidermal growth factors (EGFs) in RA^15^. Cyclooxygenase-2 (COX-2) is highly expressed in the synovial lining of RA joints because of the persistent activities of proinflammatory cytokines, such as TNF-α, IL-1β, and IL-6^16, 17^. Ribosomal S6 kinase 2 (RSK2) is an important kinase that modulates the transactivation activities of AP-1 and NF-κB, which regulate *Cox-2* gene expression in cells where growth factors and/or environmental stresses are present^18–20^, indicating the potential role of RSK2 in inflammatory diseases, such as RA. FGF receptor 3 (FGFR3) is one of four receptor tyrosine kinases that respond to FGF. Interestingly, FGFR3 activates RSK2 through tyrosine phosphorylation^21^, and its effect is associated with an enhanced MEK/ERK pathway^22, 23^. We discovered that kaempferol (3,5,7-trihydroxy-2-(4-hydroxyphenyl)-4H-1-benzopyran-4-one), a flavonoid found abundantly in edible plants, inhibited RSK2 N-terminal kinase activity by binding to the active pocket^24^, resulting in the inhibition of cell proliferation induced by EGF^25^. Recently, it was found that kaempferol inhibits RSK2, MSK1, and Src kinase activities^26, 27^. These results indicate that kaempferol might have other target proteins. The possibility of this hypothesis was suggested by the fact that the kaempferol-mediated direct inhibition of FGFR3–RSK2 signaling suppresses cell proliferation in JB6 Cl41 cells^28^. One recent study showed that the inhibition of sonic hedgehog signaling inhibits the proliferation of RA FLSs, which is associated with decreased expression of FGFR1 and FGFR3^29^.

Although a few studies have suggested that bFGF may be implicated in RA pathogenesis, its downstream signals and intracellular effects have not been well elucidated. Therefore, we investigated whether bFGF and its downstream signals contribute to the pathogenesis of RA, and how it affects RA FLS proliferation and osteoclastogenesis. To elucidate this, we studied a proliferation assay in human RA FLSs and signaling activity in RA synovium. Furthermore, an in vivo effect was confirmed through collagen-induced arthritis (CIA) mice, an experimental murine model of RA. Here, we found that kaempferol was identified as a compound that inhibits FGFR3 kinase activity in RA FLSs, resulting in significant inhibition of FLS proliferation and migration. Furthermore, kaempferol treatment in arthritis mice attenuated arthritis severity and osteoclastogenesis.

## Results

### bFGF stimulates proliferation of FLSs in humans

**Fig. 1:**
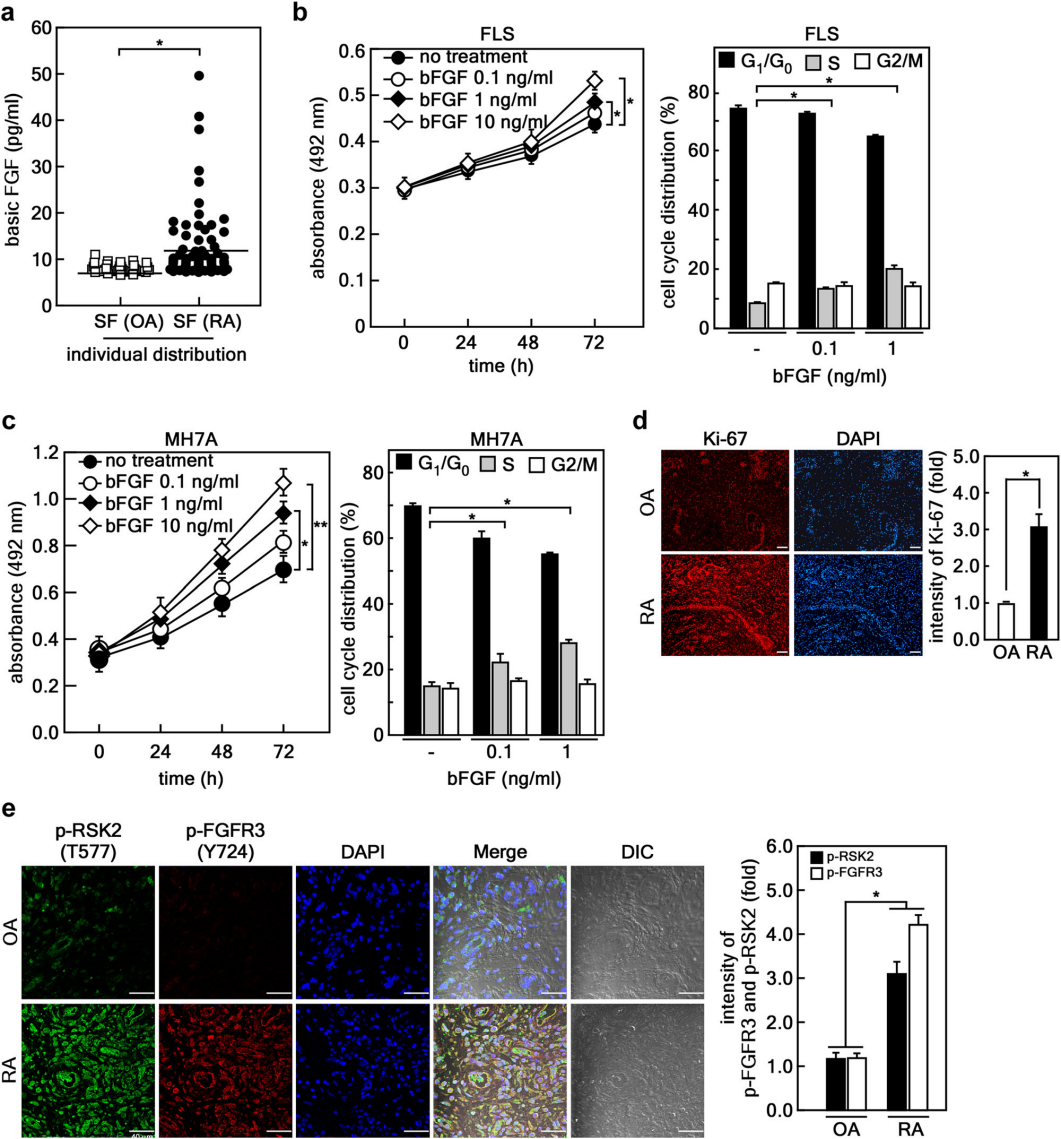
bFGF stimulates proliferation of FLSs in humans. **a** bFGF contents in SF obtained from OA patients (*n* = 31) and RA patients (*n* = 79) were measured by sandwich ELISA using specific antibodies. Each dot represents the concentration of bFGF from each patient. The median values of bFGF in the RA SF and OA SF denotes the center of interquartile range. *p < 0.01. **b**, **c** bFGF induces proliferation of human FLSs (**b**) and human MH7A (**c**) by G1/S cell-cycle transition in a dose-dependent manner compared with untreated controls. Data were obtained from three independent experiments. **d**, **e** Synovial tissues from OA patients (*n* = 5) and RA patients (*n* = 5) were analyzed to measure cell proliferation potential using Ki-67 (**d**) and the activation of RSK2 and FGFR3 using p-RSK2 (T577) and p-FGFR3 (Y724) antibodies (**e**). Scale bars, 50 μm. The photograph is a representative confocal image obtained from an immunohistofluorescence assay; the fluorescence intensities of Ki-67, p-RSK, and p-FGFR3 were normalized by DAPI intensity. The average fold change of the intensity presented in graphs was obtained from five synovial tissues from OA and RA patients. *p < 0.05; **p < 0.01

To determine whether bFGF plays a pivotal role in RA pathogenesis, we measured and compared bFGF concentrations in the SF of RA (*n* F 79) and OA (*n* = 31) patients. The mean ages of the patients with RA and OA who participated in our study were 58 and 64 years, respectively. The median duration of disease in RA patients who participated in our study was 5.8 (interquartile range [IQR], 1.5–10) years. The concentration of bFGF measured in the SF of RA patients was higher than that in the SF of OA patients (Fig. 1a). The median values of bFGF in RA SF and OA SF were 9.1 (IQR, 7.9–11.8) and 7.7 (IQR, 7.3–8.5) pg/ml, respectively. Interestingly, the bFGF level in the SF of RA patients positively correlated with SF white blood cell (WBC) and neutrophil counts (Supplementary Figure S1a and b). However, neither the Disease Activity Score 28, which is the most widely used RA activity index, nor the serum level of C-reactive protein showed any correlation with bFGF levels in SF (data not shown). Based on these findings, we examined bFGF effects on cell proliferation using both human FLSs isolated from RA patients and MH7A, a human RA synovial cell line. The results showed that bFGF-induced cell proliferation (Fig. 1b, c, left graphs) by stimulating G1/S cell-cycle transition (Fig. 1b, c, right graphs). The stimulatory effect of FLS proliferation was confirmed by immunofluorescence assay, showing that Ki-67 expression was elevated about three-fold in RA synovium compared with that in OA (Fig. 1d). Based on previous reports indicating that RSK2 is directly regulated by FGFR3^22, 28^, we examined the activation levels of RSK2 and FGFR3 in human RA and OA synovium. The results showed that the fluorescence intensities for the phospho-RSK2 (Thr577) and -FGFR3 (Tyr724) were increased in RA synovium compared with that in OA tissues (Fig. 1e).

### Involvement of FGFR3 and RSK2 in RA in humans

**Fig. 2:**
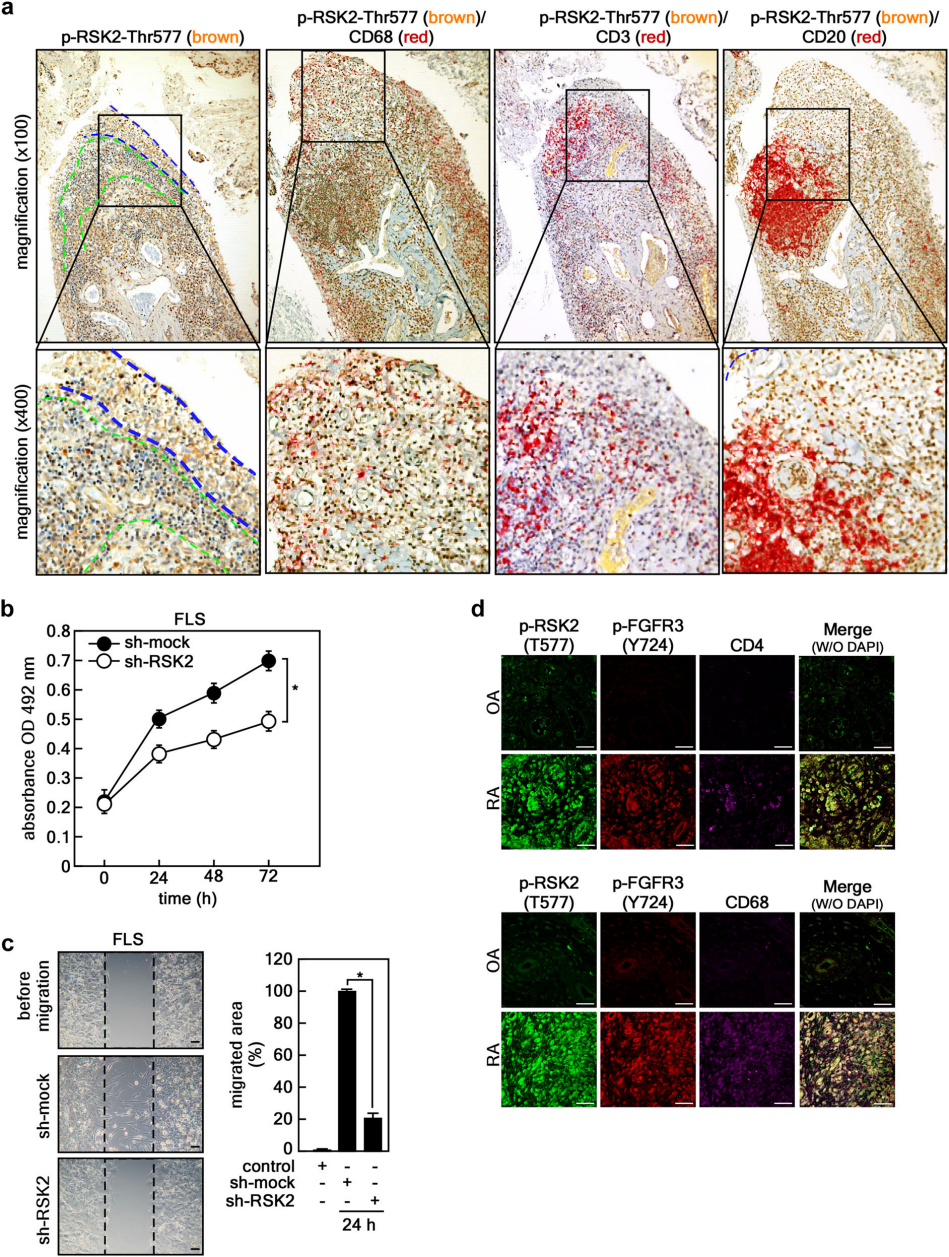
Involvement of FGFR3 and RSK2 in RA in humans. **a** Representative double immunohistochemical analysis of human RA synovial tissues (×100, *n* = 3). The tissues were hybridized with a combination of phospho-RSK2 (Thr577) antibody (shown in brown), CD68 (a macrophage marker, shown in red), CD3 (a T-cell marker, shown in red), and CD20 (a B-cell marker, shown in red), as indicated. Boxed area was magnified (×400). The area marked by the blue dotted line indicates the hyperplastic lining layer, and the area marked by the green dotted line indicates the sublining layer in synovium. **b**,** c** The knockdown effects of RSK2 on cell proliferation (**b**) and cell migration (**c**) in human RA FLSs. Cell proliferation was measured by MTS assay (**b**), and the migrated area (graph) was quantified by measuring the uncovered area of the wound using Image J (Ver. 1.6). Scale bars, 100 μm (**c**). Data were obtained from three independent experiments, and values are represented as means ± SEM. *p < 0.001 by Student’s *t*-test. **d** Representative photographs (×40) of triple immunohistofluorescence analysis of tissues obtained from OA (*n* = 5) and RA (*n* = 5) patients. p-RSK2 and p-FGFR3 were co-stained with CD4- (a T-cell marker; upper panels) or CD68- (a macrophage marker; bottom panels) specific antibodies as indicated and analyzed by confocal microscopy. Yellow color in the merged photographs indicates the coexistence of CD4^+^ T-cells or CD68^+^ macrophages in the synovial tissues of OA and RA patients, respectively. Scale bars, 40 μm

Since the RSK2 signal was found to be higher in the RA synovium compared with that in OA, we determined which cells mainly express RSK2 in RA synovium. An immunohistochemical study showed that active RSK2 was abundantly detected in the hyperplastic lining layer (indicated by the blue dotted line) and sublining layer (indicated by the green dotted line) of RA synovium (Fig. 2a, first panels). Moreover, double immunohistochemistry using antibodies against RSK2 (brown), CD68 (as macrophage markers, red), CD3 (as T-cell marker, red), and CD20 (as B-cell marker, red) macrophages were present in the hyperplastic lining layer and sublining area at a fairly high frequency (Fig. 2a, second panel), and T-cells were present in the sublining layer of lymphoid aggregates (Fig. 2a, third panel), similar to previous reports^30, 31^. CD20^+^ B cells were abundantly present in ectopic lymphoid structures with germinal center-like characteristics (Fig. 2a, fourth panel). The results further showed that some T-cells and most macrophages had activated RSK signals, but B cells did not (Fig. 2a, second to fourth panels). Since the activated RSK2 signal was highly detected in the hyperplastic lining of RA synovium, we performed RSK2 knockdown using the lentiviral shRNA expression vector. We found that RSK2 knockdown attenuated cell proliferation and migration of human RA FLSs (Fig. 2b, c). Interestingly, compared with OA tissue, RA synovium harbored higher levels of activated RSK2 (phospho-RSK2-Thr577) and FGFR3 (phospho-FGFR3-Tyr724) (Fig. 2d). Moreover, the cells expressing activated forms of RSK2 and FGFR3 were highly co-stained with CD68, and moderately with CD4 (Fig. 2d). Taken together with Figs. 1 and 2, these results imply that bFGF and its downstream FGFR3/RSK2 signaling axis might play pivotal roles in the proliferation and migration of RA FLSs and persistent inflammation in RA joints, which suggests the rationale for FGFR3/RSK2 signaling targeting in RA to inhibit the development and progression of RA.

### Kaempferol targets the kinase domain of FGFR3

**Fig. 3:**
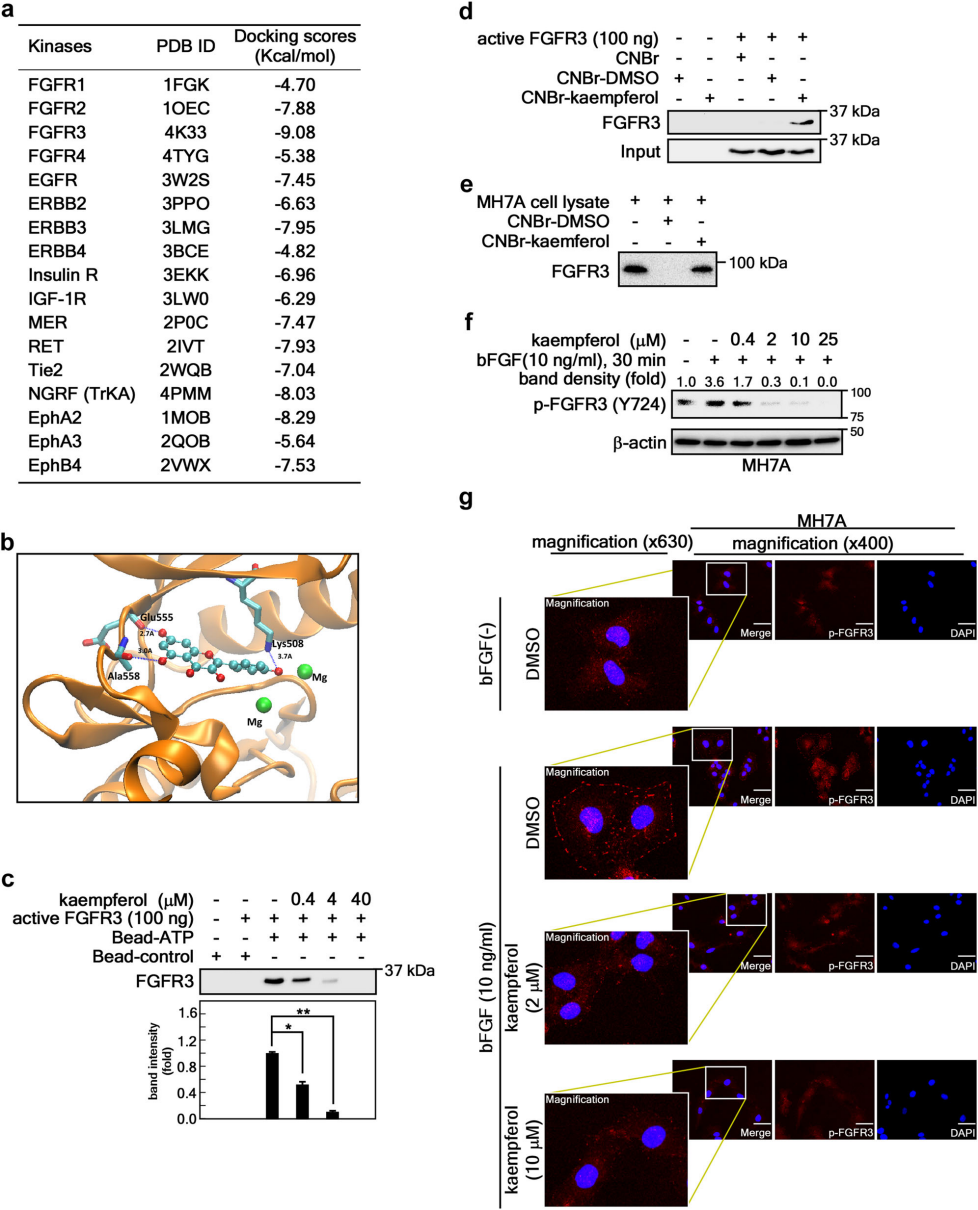
Kaempferol targets the kinase domain of FGFR3. **a** Docking scores calculated by Protein Preparation Wizard in Maestro v9.2 using kaempferol 3D structures (CID 5282102) and crystal structures of indicated RTKs obtained from the Protein Data Bank. **b** The lowest docking score of kaempferol to the active pocket of FGFR3 represents the formation of hydrogen bonds with Lys508, Glu555, and Ala558, as indicated. **c** Kaempferol competition assay. Binding of FGFR3 kinase domain to ATP-conjugated agarose beads was competed with the indicated doses of kaempferol. The graph indicates fold change of band intensity from three independent western blots of the FGFR3 pull-down assay. Data were obtained from three independent experiments, and values are represented as means ± SEM. *p < 0.01; **p < 0.001. **d**, **e** Kaempferol binding to FGFR3 was confirmed by a CNBr-kaempferol pull-down assay using a commercial FGFR3 kinase domain (**c**) and a membrane fraction of MH7A cells (**d**). The western blot is representative of three independent experiments. **f**, **g** The effects of kaempferol on bFGF-induced FGFR3 phosphorylation at Tyr724 was analyzed by western blotting (**f**) and immunocytofluorescence (**g**) using indicated p-FGFR3 specific antibodies in MH7A cells. The band intensity of western blot (**f**) was measured using Image J (Ver. 1.6), and each indicated area (**g**) in the immunocytofluorescence confocal image (×400) was magnified (×630). Scale bars, 20 μm

Based on the ability of kaempferol to inhibit both FGFR3 and RSK2 activity^28^, we investigated the therapeutic efficacy of kaempferol for RA. Before proving this, we needed to confirm the mechanisms of FGFR3 inhibition by kaempferol. First, we conducted computational reverse docking with 17 plasma membrane residential kinase structures downloaded from the Protein Data Bank (PDB; http://www.rcsb.org/pdb/home/home.do), and found that kaempferol showed the lowest stand-free energy value, with about –9.083 kcal/mol against the ATP-binding site of the FGFR3 kinase (PDB code: 4K33) (Fig. 3a). A structural analysis proposed that kaempferol can form hydrogen bonds with the backbone oxygen of Glu555 and Ala558, as well as the side chain of Lys508, with distances of about 2.7, 3.0, and 3.7 Å, respectively (Fig. 3b). This proposal was proven by a competition assay using ATP-agarose beads and an active FGFR3 kinase domain, indicating that FGFR3 kinase domain binding to ATP was decreased by the addition of kaempferol in a dose-dependent manner (Fig. 3c). Notably, the binding of kaempferol to the FGFR3 kinase domain was confirmed by a pull-down assay with cyanogen bromide (CNBr)-activated sepharose beads and a commercially active FGFR3 kinase domain (Fig. 3d), or a membrane-bound FGFR3 extracted from the membrane fraction of MH7A cells (Fig. 3e). Moreover, western blotting (Fig. 3f) and an immunocytofluorescence assay (Fig. 3g) showed that bFGF-induced FGFR3 phosphorylation was inhibited by about 50% at 0.4 μM of kaempferol treatment and almost abrogated at 2 μM of kaempferol in MH7A cells. To sum up, we discovered that kaempferol can selectively inhibit FGFR3 activity by targeting its kinase domain.

### bFGF-induced cell migration is mediated through the RSK2 signaling pathway

**Fig. 4:**
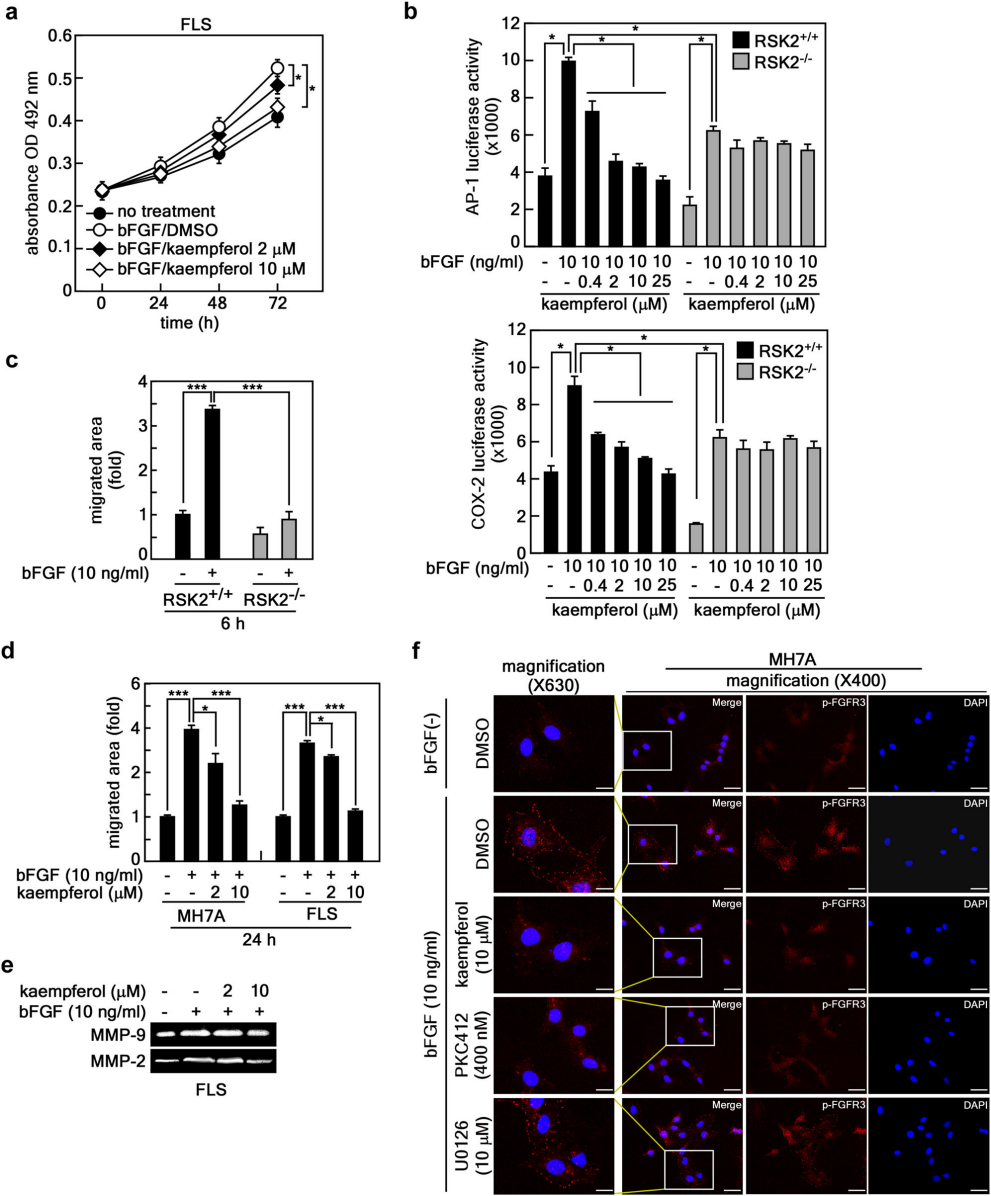
bFGF-induced cell migration is mediated through the RSK2 signaling pathway. **a** The efficacy of kaempferol on bFGF-induced human FLS proliferation was measured by MTS assay. **b** AP-1 transactivation and *Cox-2 promoter* activities were measured in RSK2^+/+^ and RSK2^−/−^ MEF by transfection of *pAP-1-luciferase* (top graph) and *pCox-2 promoter-luciferase* (bottom graph) reporter plasmids as indicated. **c** Effects of bFGF-induced cell migration in RSK2^+/+^ and RSK2^−/−^ MEFs. The migrated area was quantified by measuring the uncovered area of the wound using Image J (Ver. 1.6). **d** Efficacy of kaempferol on the cell migration of MH7A and human FLSs. The migrated area (graphs) was quantified by measuring the uncovered area of the wound using Image J (Ver. 1.6). **e** Efficacy of kaempferol on MMP-9 and MMP-2 activity was analyzed by gelatin zymography using the indicated culture supernatants of human FLSs. **f** Representative photographs for kaempferol specificity on FGFR3 phosphorylation at Tyr724 induced by bFGF stimulation in MH7A cells. PKC412, an FGFR3 inhibitor; U0126, an MEK inhibitor. Data were obtained from three independent experiments. Each indicated area in the immunocytofluorescence confocal image (×400) was magnified (×630). Scale bars, 20 μm. **a**–**d** Data were obtained from three independent experiments, and values are represented as means ± SEM. *p < 0.05; **p < 0.01; ***p < 0.001 by Student’s *t*-test

Since kaempferol targets RSK2 N-terminal kinase^24^ and the FGFR3 kinase domain (Fig. 3), we analyzed the effects of kaempferol on the proliferation and migration of human RA FLSs. Kaempferol inhibited bFGF-induced FLS proliferation in a dose-dependent manner (Fig. 4a). No cytotoxicity was observed in RA FLSs up to a concentration of 80 μM of kaempferol (Supplementary Figure S2a), similar to our previous observation^25^. Since RSK2 plays an important role in cell proliferation and migration^19, 32^, we determined the effects of kaempferol on the transactivation activities of AP-1 and NF-κB and *Cox-2* promoter activity in RSK2^+/+^ and RSK2^−/−^ mouse embryonic fibroblasts (MEFs). The results showed that bFGF-induced AP-1 transactivation activity and *Cox-2* promoter activity were decreased by kaempferol in RSK2^+/+^ MEFs, but not in RSK2^−/−^ MEFs, in a dose-dependent manner (Fig. 4b). Similar results were obtained from RSK2^+/+^ and RSK2^−/−^ MEFs in naive cell culture conditions (Supplementary Figure S2b), suggesting that the genetic depletion of RSK2 or kaempferol treatment might suppress cell migration of FLSs. As expected, the bFGF-induced cell migration shown in RSK2^+/+^ MEFs was attenuated in RSK2^−/−^ MEFs (Fig. 4c and Supplementary Figure S2c). The inhibitory effects of cell migration induced by bFGF were similarly observed by kaempferol treatment in MH7A and human RA FLSs in a dose-dependent manner (Fig. 4d). Zymography using the culture supernatant of FLSs indicated that FLS migration inhibition by kaempferol was mediated through the inhibition of MMP-9 and MMP-2 activities (Fig. 4e). Since RSK2 mediated bFGF signaling in FLS proliferation and migration (Fig. 4a–d), we needed to confirm the specificity of kaempferol on FGFR3. We found that bFGF-induced FGFR3 phosphorylation was inhibited by PKC412, a FGFR3 inhibitor, and kaempferol, but not by U0126, an MEK inhibitor (Fig. 4f). Taken together, our results suggest that the FGFR3–RSK2 signaling axis might play an important role in the proliferation and migration of FLSs, which is the most fundamental treatment target to prevent progressive joint destruction in RA.

### Kaempferol inhibits RA development in a collage-induced arthritis mouse model

**Fig. 5:**
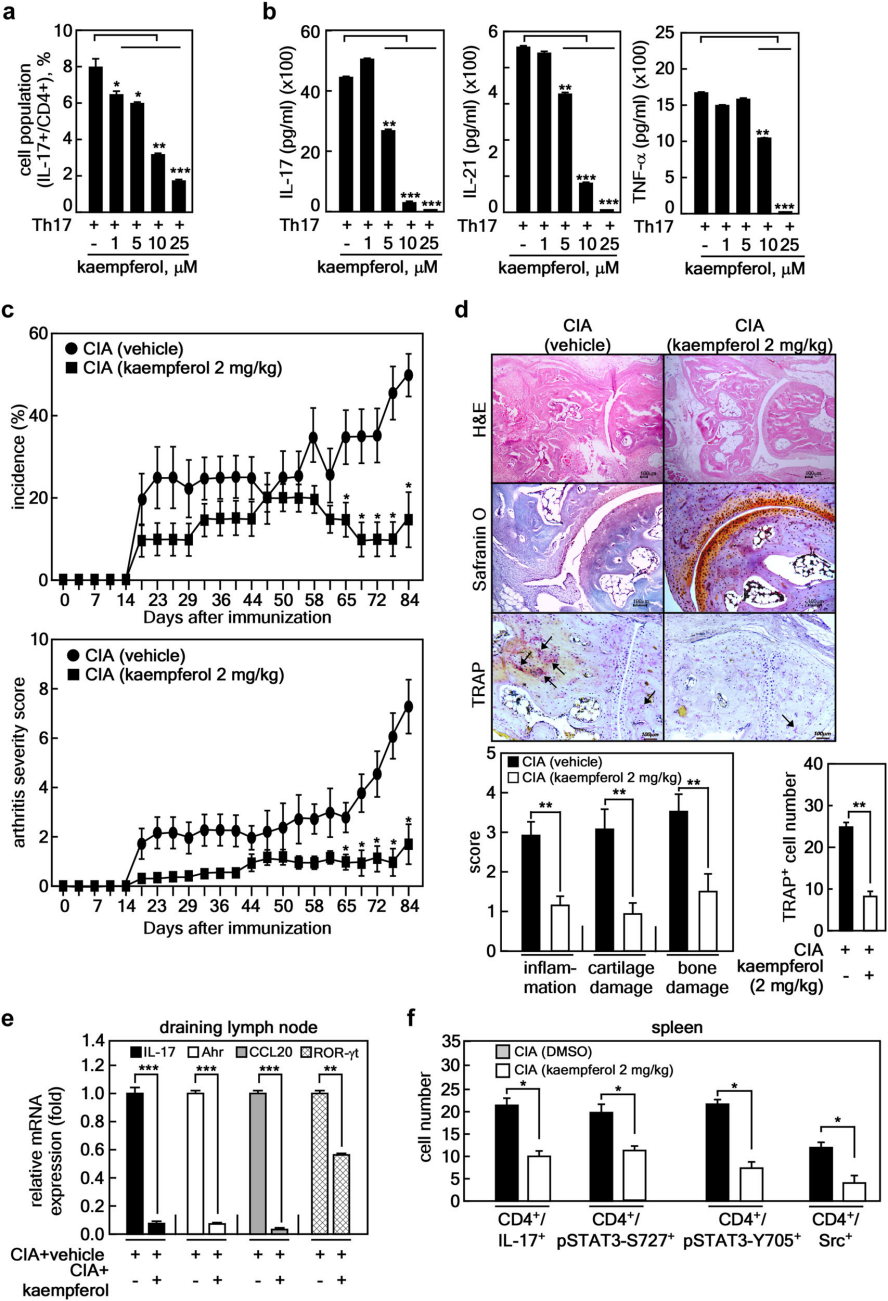
Kaempferol inhibits RA development in the collage-induced arthritis mouse model. **a**,** b** Inhibitory effects of kaempferol on Th17 lineage differentiation and inflammatory cytokine production were analyzed using mouse splenocytes. The inhibitory effects of kaempferol on Th17-polarized T-cell differentiation (**a**), secretion of IL-17 (**b**, left graph), IL-21 (**b**, middle graph), and TNF-α (**b**, right graph) were determined by counting IL-17^+^/CD4^+^-expressing T-cell cells using flow cytometry (**a**) and sandwich ELISA (**b**), respectively. Data were obtained from three independent experiments, and values are represented as means ± SEM. *p < 0.05; **p < 0.01; ***p < 0.001 vs. untreated cells. **c** Kaempferol effects on RA development in CIA mice were analyzed as described in the Materials and Methods. RA incidence (top graph) and clinical arthritis severity (bottom graph) were obtained from CIA + vehicle control group (*n* = 10) and CIA + kaempferol group (*n* = 10). Values are represented as means ± SD. Each point of the CIA + kaempferol groups was compared with the corresponding CIA + vehicle control group. *p < 0.05. **d** Representative photographs of the effects of kaempferol on RA development in CIA mice. Tissue specimens obtained from the hind paw joints of each group of mice (each *n* = 5) (**c**) at the end point of the experiment were analyzed by staining with H&E, safranin O, and TRAP, respectively. Inflammation, cartilage, bone damage, and TRAP^+^ osteoclasts were quantified as described in the Materials and Methods. Data were obtained from each group (CIA + vehicle, *n* = 5; CIA + kaempferol, *n* = 5), and values are represented as means ± SEM. **p < 0.001. **e** Inhibitory effects of kaempferol on the mRNA expression of genes involved in Th17 differentiation by real-time PCR using draining lymph node cells of CIA + vehicle and CIA + kaempferol mice, as indicated. **f** Inhibitory effects of kaempferol on the activated signaling proteins involved in the differentiation of Th17 cells were measured by immunohistofluorescence assay using the spleens of CIA + vehicle and CIA + kaempferol mice, as indicated. The spleen tissue specimens were co-stained as indicated, and the positive cells were counted using randomly photographed confocal images obtained from three different areas. **e**, **f** Data were obtained from each mouse group (CIA + vehicle, *n* = 3; CIA + kaempferol, *n* = 3), and values are represented as means ± SEM. *p < 0.05; **p < 0.01; ***p < 0.001

We investigated whether kaempferol can affect Th17 lineage differentiation (Fig. 5a, b). To investigate the effects of kaempferol under Th17 cell-polarizing conditions, isolated murine CD4^+^ T-cells were cultured in the presence of anti-CD3, anti-CD28, TGFβ, IFNγ, IL-4, and IL-6 with or without kaempferol for 72 h. We found that kaempferol decreased not only the number of IL-17-expressing CD4^+^ T-cells (Fig. 5a), but also the level of IL-17, IL-21, and TNF-α in the culture supernatant (Fig. 5b) in a dose-dependent manner. The MTT assay to investigate the cytotoxicity of kaempferol resulted in no cytotoxicity in murine CD4^+^ T-cells up to 25 μM of kaempferol (data not shown). Next, we investigated whether kaempferol suppressed inflammation and joint destruction in an experimental RA murine model (CIA). One group of mice was intraperitoneally injected with 2 mg/kg of kaempferol three times a week after type II collagen (CII) boosting immunization, and the other group was only injected with the vehicle. The results showed that kaempferol treatment in CIA mice ameliorated arthritis severity and incidence compared with vehicle-treated mice (Fig. 5c). Histological sections of hind paw joints showed that kaempferol treatment in CIA mice attenuated the severity of inflammation, cartilage damage, and bone erosion, which were investigated by hematoxylin-eosin (H&E) staining (Fig. 5d, top panels)^33^. Additionally, cartilage loss assessed by safranin O was prevented by kaempferol treatment in CIA mice (Fig. 5d, middle panels). The reduction of tartrate-resistant acid phosphate-positive (TRAP^+^, indicated by black arrow) cells in the joints of CIA+kaempferol mice indicated that osteoclastogenic activity was suppressed (Fig. 5d, bottom panels and right graph) compared with control group mice. To validate whether kaempferol treatment negatively regulated Th17 lineage differentiation in vivo, the mRNA levels of genes involved in Th17 differentiation, such as *IL-17, Ahr, CCL20*, and *ROR*γ*t*, were measured in draining lymph node cells isolated from each group of mice. The mRNA levels of the genes were lower in CIA+kaempferol mice than in vehicle-treated mice (Fig. 5e). Since the signal transducer and activator of transcription-3 (STAT3) is a pivotal transcriptional factor during the differentiation of Th17 cells from naïve CD4^+^ T-cells through *IL17a* and *IL17f* gene expression^34^, and Src is known to be capable of activating STAT3 activity by direct tyrosine phosphorylation^35, 36^, cell populations in the spleen tissue of CIA + kaempferol mice showing CD4^+^/IL-17^+^ (mainly Th17), CD4^+^/pSTAT3-S727^+^, CD4^+^/pSTAT3-Y705^+^, and CD4^+^/Src^+^ were analyzed by confocal microscope. The number of IL-17^+^/CD4^+^ cells was decreased by kaempferol treatment in CIA mice (Fig. 5f and Supplementary Figure S3a). Moreover, the numbers of CD4^+^/pSTAT3^+^ (both Ser727 and Tyr705) and CD4^+^/Src^+^ splenic T-cells were significantly decreased by kaempferol treatment compared with vehicle-treated arthritis mice (Fig. 5f and Supplementary Figure S3b). These results demonstrated that kaempferol inhibited autoimmune arthritis by suppressing Th17 cell differentiation from naïve T-cells.

### Kaempferol inhibits osteoclast differentiation

**Fig. 6:**
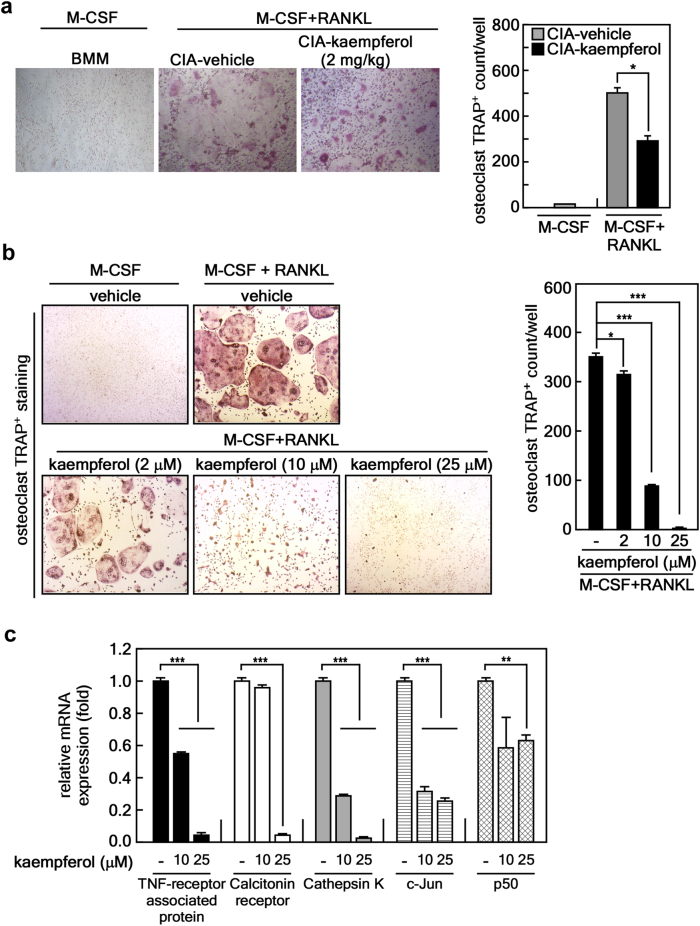

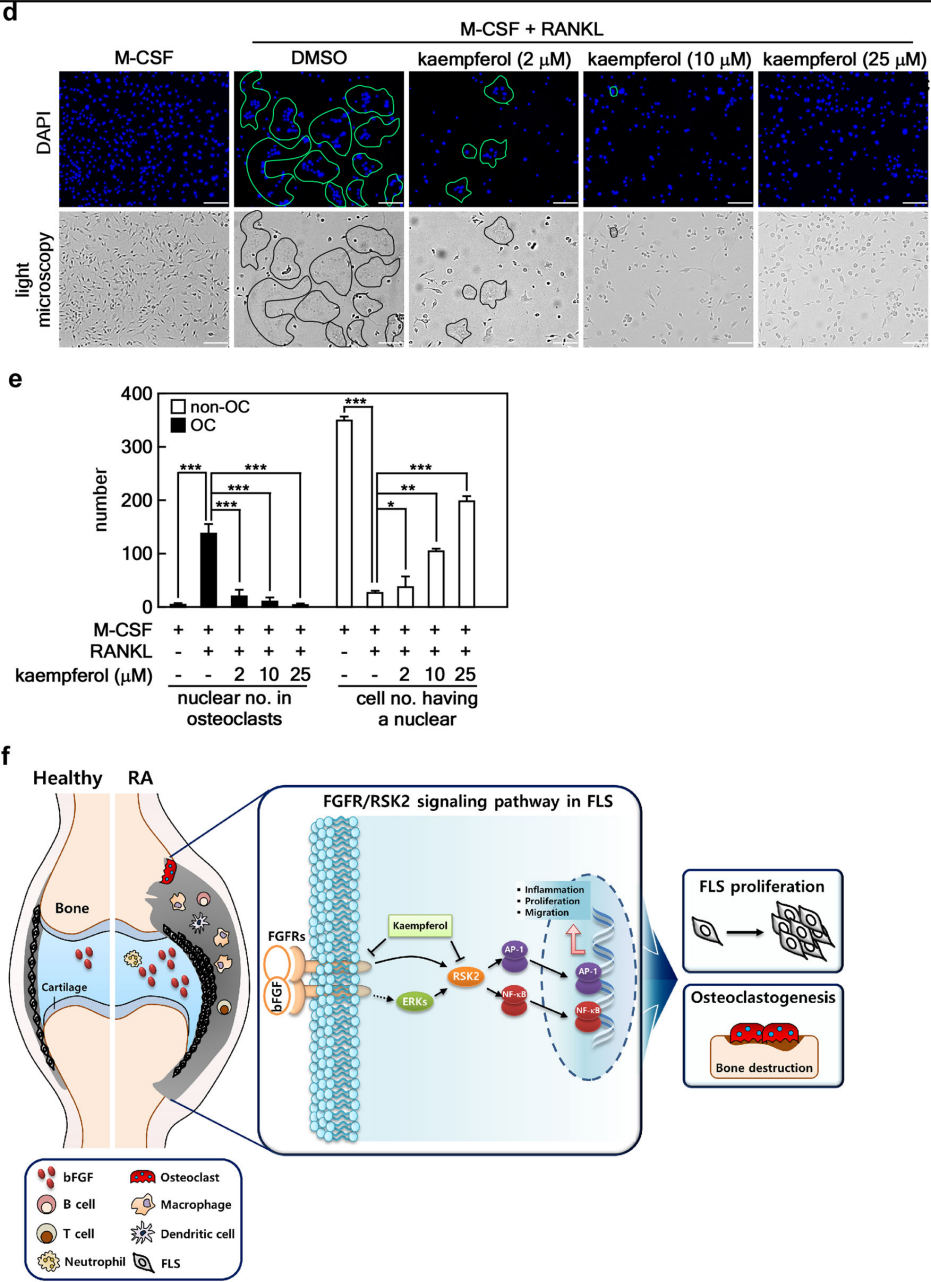
Kaempferol inhibits osteoclast differentiation. **a** Inhibitory effects of kaempferol on *ex vivo* osteoclast formation. The BMMs obtained from CIA + vehicle and CIA + kaempferol mice were analyzed in terms of the osteoclast formation induced by M-CSF or M-CSF + RANKL. TRAP^+^ osteoclasts (≥3 nuclei/TRAP^+^ cell) were counted. Photographs (×100) are representative of TRAP staining obtained from each mouse group (CIA + vehicle, *n* = 3; CIA + kaempferol, *n* = 3), and values obtained from the whole well of a 48-well plate are presented as means ± SEM. *p < 0.05. **b** Inhibitory effects of kaempferol on in vitro osteoclast formation. Naïve murine BMMs were subjected to osteoclast differentiation by combinational stimulation of kaempferol, M-CSF, and RANKL as indicated. TRAP^+^ osteoclasts ( ≥ 3 nuclei/TRAP^+^ cell) were counted. Photographs (×100) are representative of TRAP staining obtained from three independent experiments, and values obtained from the whole well of a 48-well plate are represented as means ± SEM. *p < 0.05; **p < 0.01; ***p < 0.001. **c** Inhibitory effects of kaempferol on osteoclast-specific genes. Naïve murine BMMs stimulated with M-CSF/RANKL and indicated doses of kaempferol for 4 days, and mRNA levels of indicated osteoclast-specific genes were measured by real-time PCR. Data were obtained from three independent experiments, and values are represented as means ± SEM. **p < 0.01; ***p < 0.001. **d** Representative photographs of morphological osteoclast analysis. The indicated area shows a multinucleated giant osteoclast cell body after treatment with kaempferol and M-CSF/RANL, as indicated. Scale bars, 40 μm. **e** Inhibitory effects of kaempferol on osteoclast differentiation. The total nuclear number of multinucleated (≥3 nuclei) giant cells with the phenotypic features of osteoclasts and the number of cells with a single nucleus were counted. Data were obtained from three independent experiments using a four-chamber slide, and values are represented as means ± SEM. *p < 0.05; **p < 0.01; ***p < 0.001. **f** Schematic of the signaling pathway targeted by kaempferol for the inhibition of osteoclast differentiation. bFGF-FGFR3 interaction transduces activation signaling to RSK2, resulting in hyperplasia by the induction of inflammation, FLS proliferation, and cell migration through NF-κB and AP-1. Eventually, the macrophages in synovium differentiate to bone absorbing osteoclasts. Thus, the dual targeting of kaempferol on both FGFR3 and RSK2 may prevent RA in humans

Next, we examined the effects of kaempferol on osteoclast differentiation. We found that ex vivo M-CSF/RANKL-induced differentiation of bone marrow-derived monocytes/macrophages (BMMs) isolated from CIA+kaempferol mice significantly inhibited the formation of multinucleated TRAP^+^ giant cells compared with those of CIA+vehicle mice (Fig. 6a). An in vitro study confirmed that kaempferol treatment in naïve murine BMMs significantly inhibited M-CSF- and RANKL-induced osteoclastogenesis (Fig. 6b). We further characterized the effects of kaempferol on the molecular mechanisms for osteoclast differentiation by an analysis of mRNA levels for osteoclast-specific genes, including *TRAP*, *calcitonin receptor*, *cathepsin K*, *c-Jun*, and *p50* (a member of NF-κB) (Fig. 6c). These results strongly supported the hypothesis that kaempferol inhibits osteoclast differentiation (Fig. 6c). Since osteoclasts phenotypically characterized by high TRAP activity are multinucleated cells formed by the fusion of hematopoietic lineage-derived monocytes/macrophages, we next investigated the effects of kaempferol on M-CSF/RANKL-stimulated BMM morphology and nuclear numbers in an osteoclast. The total nuclear number of multinucleated (≥3 nuclei) giant cells with the phenotypic features of osteoclasts was significantly reduced by kaempferol in a dose-dependent manner, whereas the number of cells with a single nucleus were increased (Fig. 6e). These results indicated that kaempferol inhibits the differentiation of multinucleated osteoclasts from undifferentiated BMMs with a single nucleus. Taken together, our findings indicate that bFGF/FGFR3-mediated RSK2 activation induces FLS proliferation, migration, and inflammatory responses, resulting in the induction of osteoclastogenesis. Thus, blockage of the bFGF/FGFR3/RSK2 signaling axis by kaempferol may inhibit the progressive structural damage of RA joints that is induced by overwhelming osteoclast activity.

## Discussion

In this study, the kaempferol-antagonizing bFGF/FGFR3/RSK2 axis effectively reduced clinical and histologic scores in CIA mice. The main mechanism by which kaempferol exerted its antiarthritic efficacy was the inhibition of RA FLS proliferation and migration and the significant suppression of Th17 differentiation and osteoclastogenesis. In joints, FLSs produce lubricating SF in the joint cavity, and is involved in the production of matrix components and matrix-degrading enzymes during matrix remodeling^37^. Proinflammatory factors produced by FLSs and immune cells such as activated T-cells induce the secretion of matrix-degrading enzymes and inflammatory factors that contribute to joint erosion and enhance the inflammatory cycle in RA^1–3, 38^. Since the RSK2 signaling pathway induces the transactivation activities of AP-1, and NF-κB regulates cell proliferation, cell migration, and inflammation^19, 39^ by regulation of activated T-cell 3 (NFAT3) nuclear factors and RSK2/NFAT3-mediated IL-2 promoter activity^39^, these results suggest that RSK2 plays a critical role in T-cell activation in vitro and in vivo^40^, and provide us with an opportunity to establish the hypothesis that RSK2 might be involved in RA development.

On the other hand, typical hallmarks of RA are thickening of the synovial lining and synovial hyperplasia by the enhancing fibroblast proliferation and immune cell infiltration^41^. This phenomenon eventually contributes to joint damage. Chronologically, the expression of bFGF in synovial tissues from patients with RA was detected by immunohistological staining^42^. Later, bFGF concentrations were measured in two different groups (one group with less joint damage [Larsen grade 1–3], and another with severe joint damage [Larsen grade 4–5]) of RA patients and a group of OA patients. The results indicated that the bFGF concentrations in the SF of RA patients with severe joint damage (70–73 pg/ml) were 5.5- and 20.6-fold higher than that in the SF of RA patients with less joint damage (8.4–18.9 pg/ml) and OA patients (3.6 pg/ml), respectively^6^. Based on the positive correlation between bFGF in RA SF and WBC and neutrophil counts, and the significant differences in SF bFGF concentrations according to the severity of radiographic damage, it is suggested that bFGF in RA joints can play a pivotal role in progressive joint destruction and may represent local inflammatory status in affected joints.

Osteoclast differentiation of BMMs is a complicated process governed by a variety of signaling pathways. Osteoclasts are multinucleated bone resorbing cells formed by the cytoplasmic fusion of mononuclear precursors such as macrophages. Previous reports have indicated that the chemical inhibition of the MEKs/ERKs signaling pathway using PD98059 or U0126 suppresses RANKL-induced osteoclast differentiation^40, 41^. Moreover, c-Fos, a component of AP-1, and NF-κB are well-known regulatory molecules that induce osteoclast differentiation^43, 44^. Since the inhibition of the ERKs/RSK2 signaling axis abrogated AP-1 and NF-κB transactivation activity^32^, we established our initial hypothesis that dual targeting of kaempferol on the kinase activities of FGFR3 and RSK2 might suppress osteoclastogenesis. Indeed, recent studies have shown that bFGF increases osteoclast activity in vitro and that this increasing activity is inhibited by FGFR3-deficient osteoclasts, which revealed the directly positive effect of bFGF-FGFR3 signaling on osteoclast regulation^45^. However, bFGF signaling is also known to affect osteoblast activity^46^. bFGF is expressed in osteoblasts and induces osteogenesis in a stage-specific manner in osteoblastic cells^47^. Despite these concomitant effects of bFGF on osteoblasts and osteoclasts, kaempferol showed significant anti-arthritis and anti-osteoclastic effects in vivo and in vitro.

Conclusively, our study indicated that bFGF-FGFR3 interaction stimulates FLS proliferation and cell migration mediated through both direct and indirect signaling pathways to RSK2 in the synovial tissues, resulting in the provocation of inflammatory response-macrophage infiltration. The macrophages in the synovial tissue differentiated to multinucleated osteoclasts by the stimulation of M-CSF and RANKL, resulting in RA development. Thus, the dual targeting of kaempferol on both FGFR3 and RSK2 may prevent RA development through the inhibition of osteoclast differentiation (Fig. 6f).

The efficacy of kaempferol was previously suggested by the fact that kaempferol harbored about 7 μM, 15 μM, and 25 μM of IC_50_ against RSK2, Src kinase and MSK1, respectively^24, 26, 27^. In the present study, we found that kaempferol targeted the kinase domain of FGFR3 with about 400 nM of IC_50_ in a cell culture system (Fig. 2e, f). The 400 nM IC_50_ value of kaempferol is the lowest effective concentration against known target kinases such as RSK2, MSK1, and Src. Interestingly, the kaempferol concentration in human plasma was maximally reached at 100–150 nM at 5.8 h by serving a bowl of thick endive soup (300 g; 8.65 mg kaempferol equivalent) with a slice of white bread and a glass of water after fasting from flavonoid-rich foods such as fruits, juices, green or leafy vegetables, onions, tomatoes, tea, and red wine for 48 h before the examination^48^. These results suggest that long-term daily consumption of a kaempferol-rich diet can help prevent RA development.

## Materials and methods

### Chemicals and antibodies

Chemicals utilized for molecular and cellular biology and buffer preparation were purchased from Sigma-Aldrich (St. Louis, MO, USA). Cell culture medium including Dulbecco’s Modified Eagle’s Medium (DMEM; Cat#: 10-013-CVR, Corning, New York, NY, USA), modified Eagle’s Medium (MEM; Cat#: 10-010-CVR, Corning), and supplements including penicillin and streptomycin (Cat#: 15140-122, Gibco, Waltham, MA, USA) were purchased from Life Science Technologies (Rockville, MD, USA). Antibodies for phospho-FGFR3 (Cat#: SC-33041), FGFR3 (Cat#: SC-13121), p-RSK2 T577 (Cat#: SC-16407, SC-377501), RSK2 (Cat#: SC-9986), and β-actin (Cat#: SC-69879) were purchased from Santa Cruz Biotechnology (Santa Cruz, CA, USA). Antibodies for phospho-RSK T359/S363 (Cat#: 9344), phospho-ERK1/2 (Cat#: 9106), ERK1/2 (Cat#: 9102), and Ki-67 (Cat#: 9027) were purchased from Cell Signaling Technology (Beverly, MA, USA) for the western blot analysis, immunohistochemistry, and immunocytofluorescence. Fetal bovine serum (Cat#: 26140-079, Gibco) was purchased from Life Science Technologies and heat inactivated before utilization. Human recombinant bFGF (Cat#: SRP4037, Sigma-Aldrich), dimethylsulfoxide (DMSO, Cat#: D8418, Sigma-Aldrich), and kaempferol (Cat#: ALX-385-005, Enzo, Farmingdale, NY, USA) were purchased from Sigma-Aldrich.

### Computational docking of kaempferol

Computational docking was performed to investigate the binding mode for the crucial functional groups of kaempferol and receptor tyrosine kinases, including the FGFR family. All crystal structures of the kinase domains of the receptor tyrosine kinases were obtained from the Protein Data Bank (http://www.rcsb.org/pdb/home/home.do). The crystal structure was prepared using the Protein Preparation Wizard in Maestro v9.2. Hydrogen was added consistent with a pH of 7.0., and all water molecules were removed. The structure was then minimized with a root-mean-square deviation cutoff value of 0.3 Å. The program Glide v5.7, which approximated a complete systematic search of the conformational, orientational, and positional space of the docked ligand, was used for ligand docking. The receptor grid was created with the centroid of the crystal ligand as the center of the grid. Docked ligands were treated flexibly while the kinase domain of receptor tyrosine kinase was held rigidly in the docking procedure. Flexible docking was performed using the standard precision mode. The number of poses per ligand was set to 10 in the post-docking minimization, and the best pose (with the lowest energy) was the output. The other parameters were kept at the default values.

### Cell culture

RSK2^+/+^ and RSK2^−/−^ MEFs (generously gifted from Dr. J.C. Brunung, Institute for Genetics, Center for Molecular Medicine Cologne, Cologne, Germany) were cultured in DMEM supplemented with 10% fetal bovine serum (FBS) and antibiotics at 37 °C in a 5% CO_2_ incubator. MH7A, a human RA synovial cell line obtained from the Riken cell bank (Ibaraki, Japan) through Dr. Eun-Hee Moon, Department of Bioscience and Biotechnology, Sejong University (Seoul Korea), were cultured in RPMI 1640 (Cat#: 10-040-CVR, Corning) supplemented with 10% FBS, penicillin (final concentration, 100 U/ml), streptomycin (P/S, final concentration, 0.1 mg/ml), and L-glutamine at 37 °C in a 5% CO_2_ incubator. The primary FLS cells obtained from Bucheon St. Mary’s Hospital were treated with 2 μg/ml of puromycin for 3 days to eliminate the non-infected cells.

### RSK2 knockdown in primary human FLSs

To establish the RSK2 knockdown primary human FLSs, we first produced Lenti-sh-RSK2 viral particles in HEK 293 T-cells purchased from the American Type Culture Collection. The HEK293T cells were transfected with pLenti-sh-RSK2 (Dharmacon, Lafayette, CO, USA) and packing plasmids (psPAX2 and pMD2.G from Addgene, Cambridge, MA, USA) according to the manufacturer’s suggested protocols. The medium containing viral particles was collected at 24 h and 48 h after transfection, filtered with 0.45 μM filters (Cat#723-2545, Thermo Fisher Scientific, Waltham, MA, USA), and used to infect human FLSs with 4 μg/ml of polybrene. After 48 h incubation, non-infected primary FLSs were eliminated by treatment of 2 μg/ml of puromycin (Cat#A111308, Thermo Fisher Scientific) for 3 days. The survived cells were immediately utilized to the cell proliferation and cell migration assays.

### Western blotting

Each equal amount of protein was resolved by 8–10% sodium dodecyl sulfate-polyacrylamide gel electrophoresis (SDS-PAGE) and transferred onto polyvinylidene difluoride (PVDF, Merck Millipore Ltd, Burlington, MA, USA) membranes. The membranes were then incubated in a blocking buffer containing 5% skim milk/1 × PBS and probed with specific antibodies as indicated, respectively. The proteins were visualized by an enhanced chemiluminescence detection system (Amersham Biosciences, Piscataway, NJ, USA) using a Chemidoc XRS imager system (Bio-Rad Laboratories, Hercules, CA, USA).

### MTS assay

To measure cell proliferation, MH7A (9 × 10^3^ cells/cm^2^), primary FLSs (9 × 10^3^ cells/cm^2^), or RSK2 knockdown FLSs (9 × 10^3^ cells/cm^2^) were seeded into 96-well plates in 100 μl of cell culture medium and incubated for 2 h at 37 °C in a 5% CO_2_ incubator. At 0 h, the absorbance was measured at optical densities of 492 nm and 690 nm using the 3-(4,5-dimethylthiazol-2-yl)-5-(3-carboxymethoxyphenyl)-2-(4-sulfophenyl)-2H-tetrazolium (MTS)-based CellTiter 96^®^ Aqueous One Solution according to the manufacturer’s instructions (Promega, Madison, WI, USA). Briefly, 20 μl of the MTS solution was added to the well, followed by incubation for 1 h at 37 °C in a 5% CO_2_ incubator. The reaction was stopped by adding 25 μl of 10% SDS solution to each well, and the absorbance was measured immediately at 492 and 690 nm. The inhibition of cell proliferation by kaempferol was evaluated by comparing the absorbance of the samples to a vehicle (DMSO)-treated control group over 96 h at 24 h intervals.

### Th17 cell differentiation and flow cytometry analysis

To measure the cell-cycle, FLS (2.5 × 10^3^ cells/cm^2^) were seeded into 60-mm-diameter dishes and cultured overnight at 37 °C in a 5% CO_2_ incubator. To examine the cell-cycle transition by bFGF stimulation, primary FLS obtained from RA patients were treated with the indicated doses of bFGF for 24 h in complete cell culture medium. The cells were trypsinized, fixed, and then stained with propidium iodide (20 μg/ml) for 15 min at 4 °C. The cell-cycle distribution was measured by flow cytometry (BD FACSCalibur™ flow cytometer, Franklin Lakes, NJ, USA). To measure the effects of kaempferol on Th17 differentiation, splenic CD4 + T-cells were stimulated with plate-bound anti-CD3 mAb (0.5 μg/ml; BD Biosciences, San Jose, CA), soluble anti-CD28 mAb (1 μg/ml; BD Biosciences), anti-IFN-γ Ab (2 μg/ml; R&D Systems, Minneapolis, MN), anti-IL-4 Ab (2 μg/ml; R&D Systems), recombinant TGF-β (2 ng/ml; R&D Systems), and recombinant IL-6 (20 ng/ml; R&D Systems) for 3 days. The cells were treated with an indicated dose of kaempferol for 3 days. Cells were stained with various combinations of fluorescent antibodies against CD4 and IL-17 (eBioscience, San Diego, CA, USA). Prior to intracellular staining, the cells were restimulated for 4 h with phorbol myristate^12^ acetate (25 ng/ml) and ionomycin (250 ng/ml) in the presence of GolgiStop (BD Bioscience). The cells were permeabilized and fixed Cytofix/Cytoperm (BD Bioscience), as per the manufacturer’s instructions and further stained with anti-IL-17 or anti-Foxp3 Flow cytometry was conducted on FACSCalibur flow cytometer (BD Biosciences).

### Wound healing assay

MH7A (3 × 10^5^ cells/cm^2^), RSK2^+/+^ (3 × 10^5^ cells/cm^2^), RSK2^−/−^ MEFs (3 × 10^5^ cells/cm^2^), and FLSs (3 × 10^5^ cells/cm^2^) were seeded into culture inserts (Cat#: 80209, Ibidi GmbH, Martinsried, Germany), cultured, and starved for 24 h. Next, the culture inserts were removed after 2 h of mitomycin-C treatment to stop cell proliferation. The cells were pretreated with the indicated doses of kaempferol for 30 min and co-treated with 1 or 10 ng/ml of bFGF in addition to the indicated doses of kaempferol. Representative images (×40) of cell migration for wound healing were captured at 0, 6, and 24 h using an ECLIPSE Ti inverted fluorescence microscope (NIKON Instruments Korea, Gangnam, Seoul, Korea). The migrated area was measured using Image J (NIH Image J Ver. 1.6, Bethesda, MD, USA).

### Reporter gene assay

RSK2^+/+^ (5 × 10^3^ cells/cm^2^) and RSK2^−/−^ (5 × 10^3^ cells/cm^2^) MEFs were transiently transfected with 400 ng each of a *pAP-1-luciferase*, *pNF-κB-luciferase*, or *pCOX-2-promoter* luciferase reporter plasmid with 20 pg of the *phRL-SV40* Renilla luciferase reporter plasmid in 24-well plates. The cells were cultured for 24 h and then starved in serum-free medium for 16 h. The cells were pretreated with the indicated doses of kaempferol for 30 min, and then co-treated with bFGF (10 ng/ml) and the indicated doses of magnolin for 24 h. The cells were disrupted, and firefly luciferase activity was measured using a VICTOR X3 (PerkinElmer, Waltham, MA, USA). Firefly luciferase activity was normalized by Renilla luciferase activity to equalize the transfection efficiency.

### Gelatin zymography

MMP-2 and MMP-9 activities were evaluated by gelatin zymography using the cell culture supernatants. Briefly, FLSs (2 × 10^4^cells/cm^2^) were seeded into 60-mm-dishes, cultured, and treated with the indicated doses of bFGF and kaempferol for 24 h. The culture supernatants were then harvested, and 20 μg of protein from each sample was loaded on a polyacrylamide gel containing 0.2% gelatin. The gel was washed by 2.5% Triton X-100 buffer for 20 min, and then incubated for 24 h at 37 °C in a renaturing buffer (50 mM Tris-HCl, pH 7.5, 10 mM CaCl_2_, 1 μM ZnCl_2_, 0.01% NaN_3_). The gels were stained with Coommassie Brilliant Blue and destained in methanol/acetic acid.

### Pull-down assay

CNBr-activated sepharose 4B beads (Cat#: 71-7086-00 AF, GE Healthcare, Little Chalfont, 9NA, UK) were activated according to the manufacturer’s suggested protocol. Briefly, CNBr-sepharose 4B beads (0.3 g) were suspended in a final concentration of 1 mM HCl (30 ml) for 5 min by rotation, and then washed with 30 ml of 1 mM HCl three times. Kaempferol (40 mM) dissolved in 100% DMSO was combined with coupling buffer (0.1 M of NaHCO_3_, pH 8.3, containing 0.5 M of NaCl) to achieve the final concentration of 10 μM. The activated CNBr-sepharose beads and kaempferol were mixed for 1 h at room temperature, and excess kaempferol was washed with at least five volumes of coupling buffer. The remaining active groups of CNBr-beads were blocked by adding the blocking buffer (0.1 M of Tris-HCl, pH 8.0) for 2 h standing. The CNBr-kaempferol beads (50% slurry) were aliquoted and stored at 4 °C until utilized. The binding between FGFR3 and kaempferol was examined by affinity chromatography. The kinase domain of active FGFR3 (100 ng) or the total membrane fraction protein (500 μg) was incubated with 30 μL of CNBr-kaempferol beads (50% slurry) for 2 h or overnight at 4 °C. The beads were washed three times and suspended in 20 μL of 1 × SDS sample buffer. Bound proteins were resolved by 10% SDS-PAGE and visualized by western blot using total-FGFR specific antibodies and horseradish peroxidase (HRP)-conjugated secondary antibodies.

### Kaempferol/ATP competition assay

Active FGFR3 (100 ng) and each indicated dose of kaempferol were combined and preincubated in binding buffer (10 mM Tris-HCl, 50 mM KCl, 5 mM MgCl_2_, 2 mM DTT, 0.01% NP-40) overnight at 4 °C, and 25 μl of ATP-agarose or control beads (50% slurry, Innova Bioscience, Cambridge, UK) was added and incubated for 2 h at 4 °C. The beads were then washed with binding buffer and mixed with 20 μl of SDS sample buffer, and FGFR3 bound ATP-agarose beads were then visualized by western blotting using total-FGFR3-specific antibodies and HRP-conjugated secondary antibodies. Band intensity was measured using a densitometry computer program (NIH Image J).

### Immunocytofluorescence assay

MH7A cells (1 × 10^4^ cells/cm^2^) were seeded into four-chamber culture slides and cultured for 12 h. The cells were then starved with FBS-free MEM for 16 h, pretreated with the indicated doses of kaempferol, U0126, and PKC412 for 30 min, and then co-treated with bFGF (10 ng/ml). The cells were fixed with 4% formalin, permeabilized with 0.5% Triton X-100/1 × PBS, blocked with 1% of BSA, and hybridized with anti-rabbit phospho-FGFR3 Tyr724-specific antibodies overnight at 4 °C in a humidified chamber. The FGFR3 proteins were visualized by hybridization with secondary antibodies conjugated with anti-rabbit-Alexa-568 (Cat#: A11036, Thermo Fisher Scientific) under an ECLIPSE Ti inverted fluorescence microscope (NIKON Instruments Korea).

### Approval statements of human subjects and animal studies

Human subject experiments using synovial fluids were obtaind from 31 OA patients (27 women and four men met the ACR criteria)^35^ and 79 RA patients (63 women and 16 men met the ACR criteria)^36^ who visited the outpatient department at the Division of Rheumatology, Bucheon St. Mary’s Hospital. WBC and neutrophil counts in the SF of OA and RA patients were calculated using a Neubauer hemocytometer and Wright’s stain, respectively. The amount of bFGF in SF was measured using an ELISA kit (R&D Systems, Minneapolis, MN, USA) according to the manufacturer’s instructions. SF samples were stored at –80 °C until analysis. RA and OA synovial tissues were obtained from patients with RA or OA undergoing total joint replacement surgery. RA FLSs were isolated by enzymatic digestion of synovial tissues obtained from RA patients undergoing total knee replacement surgery. Human experiments were approved by the Institutional Review Board (IRB) of human subjects at Bucheon St. Mary’s Hospital (approval number: HC14TISI0070), The Catholic University of Korea, and conducted in accordance with IRB guidelines and regulations. All patients were informed and gave their written consent, and the study was performed in accordance with the Helsinki II Declaration. Synovium samples were fixed in 4% paraformaldehyde solution overnight at 4 °C, dehydrated with alcohol, washed, embedded in paraffin, and sectioned into thick slices. On the other hand, all of the animal experiments, including CIA induction and kaempferol administration, were approved by the Institutional Animal Care and Use Committee (IACUC) at the Catholic University of Korea (approval number: 2013-0020-01) under specific pathogen-free conditions in accordance with IACUC guidelines and regulations.

### Measurement of bFGF in synovial fluids of OA and RA patients

The concentration of bFGF in the synovial fluid of each RA and OA patients were measured using a commercial sandwich ELISA kit (Cat#: DFB50, R&D Systems). To eliminate the influence of rheumatoid factor on the cytokine level in ELISA, all synovial fluids were precleared using a commercial reagent to block heterophilic antibodies (Cat#: 500-11-001, HeteroBlock; Omega Biologicals Inc., Bozeman, MT, USA).

### Culture of human FLSs

Synoviocytes were isolated by enzymatic digestion of synovial tissue specimens. The tissue samples were minced into 2–3 mm pieces and treated for 4 h with 4 mg/ml type II collagenase (Worthington, Freehold, NJ) in DMEM at 37 °C in 5% CO_2_. Dissociated cells were centrifuged at 500× *g*, resuspended in DMEM supplemented with 10% fetal calf serum, 2 mM L-glutamine, 100 units/ml penicillin, and 100 ng/ml streptomycin, plated in 75-cm^2^ flasks, and incubated overnight. The non-adherent cells were then removed, and the adherent cells were cultivated in DMEM supplemented with 10% fetal calf serum. Synoviocytes from passages 4–8 were used in each experiment. The cells were morphologically homogeneous and exhibited the appearance of synovial fibroblasts, with typical bipolar configuration under inverse microscopy. The cells were seeded in six- or 24-well plates, eight-well chamber slides, or 100-mm culture dishes supplemented with 10% fetal bovine serum.

### Immunohistofluorescence assays

To determine the expression of phospho-RSK2 and phopsho-FGFR3 in synovial tissues from RA and OA patients, the paraffin-invaded human synovial slices (5-μm) were deparaffinized by incubation at 60 °C for 2 h. The deparaffinized slides were rehydrated, unmasked by soaking in boiling 10 mM sodium citrate buffer (pH 6.0) for 10 min, and allowed to cool to room temperature gradually or treated with pepsin for 30 min. The slides were then blocked with 5% goat serum in 1 × PBX/0.5% Triton X-100 for 1 h at RT, and then hybridized with the indicated antibodies, phospho-FGFR3 (Y724) (1:50), phospho-RSK2 (T577) (1:50), CD68 (1:50) and CD4 (1:50), in 1 × PBS/0.5% Triton X-100 buffer overnight at 4 °C. The slides were washed and hybridized with secondary antibodies conjugated with Alexa-488 (Cat#: A11055, Bio-Rad Laboratories), −568 (Cat#: A11036, Bio-Rad Laboratories), or −647 (Cat#: A21235, Bio-Rad Laboratories) for 2 h at RT in the dark as indicated. Image stacks were captured using laser scanning confocal microscopy (LSM 710, Carl Zeiss Korea Co. Ltd., Seoul, Korea). To analyze the effects of kaempferol on signaling pathways for immune responsiveness in CIA-kaempferol treatment, the spleen tissue was snap-frozen in liquid nitrogen and stored at −70 °C. The spleen tissue sections (5-μm) were fixed in acetone and co-hybridized with a specific antibody using Fluorescein isothiocyanate-labeled anti-CD4 (Cat#: 553046, BD Biosciences, San Jose, CA, USA) and phycoerythrin (PE)-labeled anti-IL-17 (Cat#: 559502, BD Biosciences), PE-labeled anti-Src antibodies (Cat#: ab47405, Abcam, Cambridge, UK), PE-labeled anti-phospho-STAT3-Y705 (Cat#: 612569, BD Biosciences), or -S727 antibodies (Cat#: 558557, BD Biosciences). Image stacks were captured using laser scanning confocal microscopy (LSM 710, Carl Zeiss Korea Co. Ltd.). Positive cells were counted manually at a higher magnification (projected on a screen) by four individuals, and the results were expressed as means ± standard deviation.

### Double immunohistochemistry of human RA tissues

To determine cell types encountering phospho-RSK2 in RA tissues in RA patients, the RA tissues were fixed in formalin and embedded in paraffin, and then slices with 3-μm paraffin sections were prepared. The sections were deparaffinized, and then pretreated with cell conditioning solution (CC1, Ventana, Tucson, AZ, USA) and UV irradiation to abrogate the endogenous hydroperoxidase activity. First, the sections were hybridized with primary antibodies, including CD3 (Cat#: A0452, 1:100 dilution, DAKO Korea, Sonpa-gu, Seoul, Korea), CD20 (Cat#: M0755, 1:100 dilution, DAKO), and CD68 (Cat#: M0814, 1:100 dilution, DAKO), as indicated for 32 min and with AP-conjugated secondary antibody for 8 min. The proteins were visualized using an Ultra View Universal Red detection kit (Cat#: 760-501, Ventana). Second, the sections were hybridized with phospho-RSK2-Thr577 (Cat#: sc16407, 1:100 dilution, Santa Cruz Biotechnology) for 32 min with HRP-conjugated secondary antibody for 8 min. RSK2 proteins were visualized by colorimetric detection using 3,3-diaminobenzidine with H_2_O_2_. Finally, the sections were counterstained with Hematoxylin II (Ventana) for 4 min and Bluing Reagent (Ventana) for 4 min. The sections were observed under light microscope (BX50, Olympus, Japan).

### Kaempferol effects on the IL-17, IL-21 and TNF-α production in differentiated Th17 cells

The examine the effect of kaempferol on the IL-17, IL-21, and TNF-α production in differentiated T-cells, culture supernatants were obtained from Th17-polarized differentiated T-cells by the treatment of a indicated dose of kaempferol. The levels of IL-17, IL-21, and TNF-α in the supernatants of murine splenocyte cultures were measured sandwich enzyme-linked immunosorbent assay (Cat#:MAB721, Cat#:841338, AF-410-NA, R&D Systems, respectively). Horseradish peroxidase-avidin (R&D Systems) was used for color development Absorbance was measured at 405 nm on an ELISA microplate reader (Molecular Devices, Sunnyvale, CA, USA).

### Kaempferol effects on arthritis inhibition in CIA mouse model

DBA/J1 mice (20 male, 6 weeks old) purchased from Orient Bio Inc. (Guro-gu, Seoul, Korea) were acclimated for 1 week with on a 12-h dark/light cycle and allowed food and water ad libitum. To induce arthritis, DBA/1 J mice were intradermally injected at the base of the tail with 100 μg of chicken CII emulsified in complete Freund’s adjuvant (1:1 w/v; Chondrex, Redmond, WA, USA) and boosted intradermally 14 days later. Arthritic score measurements were performed as follows: 0 = no joint swelling; 1 = slight edema and erythema limited to the foot or ankle; 2 = slight edema and erythema from the ankle to the tarsal bone; 3 = moderate edema and erythema from the ankle to the tarsal bone; and 4 = edema and erythema extending from the ankle to the entire leg, with severe swelling of the wrist or ankle. The final arthritis score was calculated as the sum of scores from all four legs, which were assessed by three independent observers with no knowledge of the experimental groups. The mice were randomly divided two groups (vehicle and kaempferol injection groups). Kaempferol (2 mg/kg) dissolved in 10% DMSO was administered through i.p. injection three times a week after induction of arthritis for next 70 days. The onset and severity of arthritis were determined by three independent observers, based on the previously described scoring system^49^. The mice were observed twice a week for the onset and severity of joint inflammation for up to end point (day 84) after the initial immunization. Before being sacrificed at 84 days after CIA induction by cervical dislocation, the mice were anesthetized using 2–3% isoflurane. At end point, the mice were scarified and hind joint tissues, spleen, and tibias and femurs were harvested for the further studies. The hind joint tissues from CIA-vehicle and CIA-kaempferol groups were subject to histopathological examination such as scoring of inflammation, destruction of cartilage, and bone damage according to published criteria^50, 51^ by Hematoxylin-Eosin (H&E) staining and safranin O staining.

### Histopathological analysis of mouse arthritic joint tissues

The ankle joint specimens (5 μm) obtained from CIA and CIA-kaempferol administered mice were fixed with 4% paraformaldehyde, decalcified in a histological decalcifying agent (Calci-Clear Rapid, Cat#: HS105, National Diagnostics-Chayon Laboratories, Gangnam-gu, Seoul, Korea), and embedded in paraffin. The tissue slices were then stained with H&E and safranin O to detect proteoglycans in the joint tissues. The H&E-stained tissue slices were scored for inflammation and bone erosion as previously reported^33, 51^, and cartilage damage was determined using safranin O staining. The extent of cartilage damage was scored as described previously^33, 51^. Mouse joint tissue was fixed in 10% formalin and decalcified in EDTA bone decalcifier, and ankle joints were processed for paraffin embedding, from which 7-μm-thick tissue sections were prepared. Sections were stained for TRAP using the Leukocyte Acid Phosphatase kit (Sigma-Aldrich) according to the manufacturer’s protocol. TRAP + multinucleated cells with ≥3 nuclei were counted as osteoclasts. All histological assessments were performed by two independent blinded observers.

### Ex vivo and in vitro osteoclastogenesis

For the ex vivo osteoclastogenesis experiment, BMMs obtained from CIA-vehicle and CIA-kaempferol mice were isolated from the tibias and femurs of the mice by flushing the bone marrow cavity with α-minimum essential medium (α-MEM, Cat#: LM008-01, Wel gene, Gyeongsangbuk-do, Gyeongsna-si, Korea). The cells were centrifuged, red blood cells were removed using the ACK buffer (0.15 M NH_4_Cl, 1.0 mM KHCO_3_, 0.1 mM Na_2_EDTA), and the cells were then plated in six-well plates (Cat#: 140675, Thermo Scientific Inc., MA, USA) in α-MEM for 12 h. The floating cells were collected, seeded in 48-well plates (1 × 10^5^ cells/cm^2^) or four-chamber slides (1 × 10^5^ cells/cm^2^, Cat#: T460-27, Waltham, MA, USA), and cultured in α-MEM supplemented with 10 ng/ml of M-CSF (R&D Systems) for 3 days for differentiation into macrophage-like osteoclast precursor cells. The non-adherent cells were washed out, and the remaining osteoclast precursor cells were cultured in α-MEM supplemented with 10 ng/ml of M-CSF and 50 ng/ml of RANKL (PeproTech, Rocky Hill, NJ, USA) for 4 days to generate osteoclasts. The differentiated osteoclasts were visualized by TRAP staining. For in vitro osteoclastogenesis, non-treated DBA/J1 mice were utilized to isolate and induce the osteoclastogenesis as described above. The osteoclast precursor cells were cultured in α-MEM supplemented with 10 ng/ml of M-CSF and 50 ng/ml of RANKL either with or without kaempferol (10 μM) for 4 days to generate osteoclasts. The differentiated osteoclasts were visualized by TRAP staining. The tibias and femurs were examined to determine the ex vivo osteoclastogenesis induced by the macrophage-colony stimulating factor (M-CSF; Cat#: 300-25, PeproTech)/receptor activator of the NF-κB ligand (RANKL; Cat#: 310-01 C, PeproTech).

### Quantitative real-time polymerase chain reaction (PCR)

Total RNA was extracted using TRIzol Reagent (Molecular Research Center, Cincinnati, OH, USA) according to the manufacturer’s suggested protocol. Complementary DNA was synthesized using the Transcriptor First Strand cDNA Synthesis Kit (Roche Applied Science, Cat# 04 896 866 001, Mannheim, Germany)) and random hexamer primers. A Light-Cycler 2.0 instrument (software version 4.0; Roche Diagnostics) was used for PCR amplification. All reactions were performed using LightCycler FastStart DNA Master SYBR Green I mix (Takara) following the manufacturer's instructions. The primers for the quantitative real-time PCR were summarized in Table 1.

**Table 1:** Primer list to be utilized for quantitative real-time PCR

IL-17	sense	5′-ACC TCA CAC GAG GCA CAA GT-3′
	antisense	5′-CCC AAC AGC TGG AAT AGA GC-3′
Ahr	sense	5′- AGC AGC TGT GTC AGA TGG TG-3′
	antisense	5′-CTG AGC AGT CCC CTG TAA GC-3′
CCL20	sense	5′-CAG CTG TTG CCT CTC GTA CA-3′
	antisense	5′-CAC CCA GTT CTG CTT TGG AT-3′
ROR-yt	sense	5′-TGT CCT GGG CTA CCC TAC TG-3′
	antisense	5′-GTG CAG GAG TAG GCC ACA TT-3′
TRAP	sense	5′-CTG TGG GCT TTA AGG ACA GC-3′
	antisense	5′-ACA TAG CCC ACA CCG TTC TC-3′
Integrin β3	sense	5′-CTG TGG GCT TTA AGG ACA GC-3′
	antisense	5′-GAG GGT CGG TAA TCC TC-3′
MMP9	sense	5′-CTG TCC AGA CCA AGG GTA CAG CCT-3′
	antisense	5′-GAG GTA TAG TGG GAC ACA TAG TGG-3′
Calcitonin receptor	sense	5′-CGG ACT TTG ACA CAG CAG AA-3′
	antisense	5′-AGC AGC AAT CGA CAA GGA GT-3′
Cathepsin K	sense	5′-CAG CAG AGG TGT GTA CTA TG-3′
	antisense	5′-GCG TTG TTC TTA CGA GC-3′
c-Jun	sense	5′-GCA GAA AGT CAT GAA CCA CG-3′
	antisense	5′-TCG CAA CCA GTC AAG TTC TC-3′
p50 (a component of NF-κB)	sense	5′-GTC TCT GGG GGT ACC ATC AAA G-3′
	antisense	5′-AGG ATG TCT CCA CAC CAC TGT-3′
β-actin	sense	5′-GAA ATC GTG CGT GAC ATC AAA G-3′
	antisense	5′-TGT AGT TTC ATG GAT GCC ACA G-3′

### Statistical analysis

Data are presented as the mean ± SEM. The Mann–Whitney *U* test or Student's *t*-test was used for comparing values between two groups. One-way analysis of variance followed by Bonferroni’s post hoc test was used to compare the differences between three or more groups. To assess the Gaussian distribution and the equality of variance, the Shapiro–Wilk test and Levene test were used, respectively. Differences between arthritis incidences at a given time point were analyzed by the χ2 contingency analysis. The program used for the statistical analysis was the SPSS statistical software package, standard version 16.0 (SPSS, Chicago, IL, USA). *P*-values < 0.05 (two-tailed) were considered significant.

## Electronic supplementary material

Supplementary Information(DOC 5497 kb)
