# Supplementary material for: Kaempferol targeting on the fibroblast growth factor receptor 3-ribosomal S6 kinase 2 signaling axis prevents the development of rheumatoid arthritis
Source: Cell Death Dis. 2018 Mar 14;9(3):401. doi: 10.1038/s41419-018-0433-0 (PMC5851988; doi:10.1038/s41419-018-0433-0)

**[Supplementary Information]**

**Kaempferol targeting on the fibroblast growth factor receptor 3-ribosomal S6 kinase 2 signaling axis prevents rheumatoid arthritis development**

Cheol-Jung Lee, Su-Jin Moon, Jeong-Hee Jeong, Sangbae Lee, Mee-Hyun Lee, Sun-Mi Yoo, Hye Suk Lee, Han Chang Kang, Joo Young Lee, Weon Sun Lee, Hee-Jin Lee, Eun-Kyung Kim, Joo-Yeon Jhun, Mi-La Cho, Jun-Ki Min, and Yong-Yeon Cho

**Contents**

1. Supplementary Figures S1-S3

2. Supplementary Western Blot Data

**1. Supplementary Figures S1-S3**


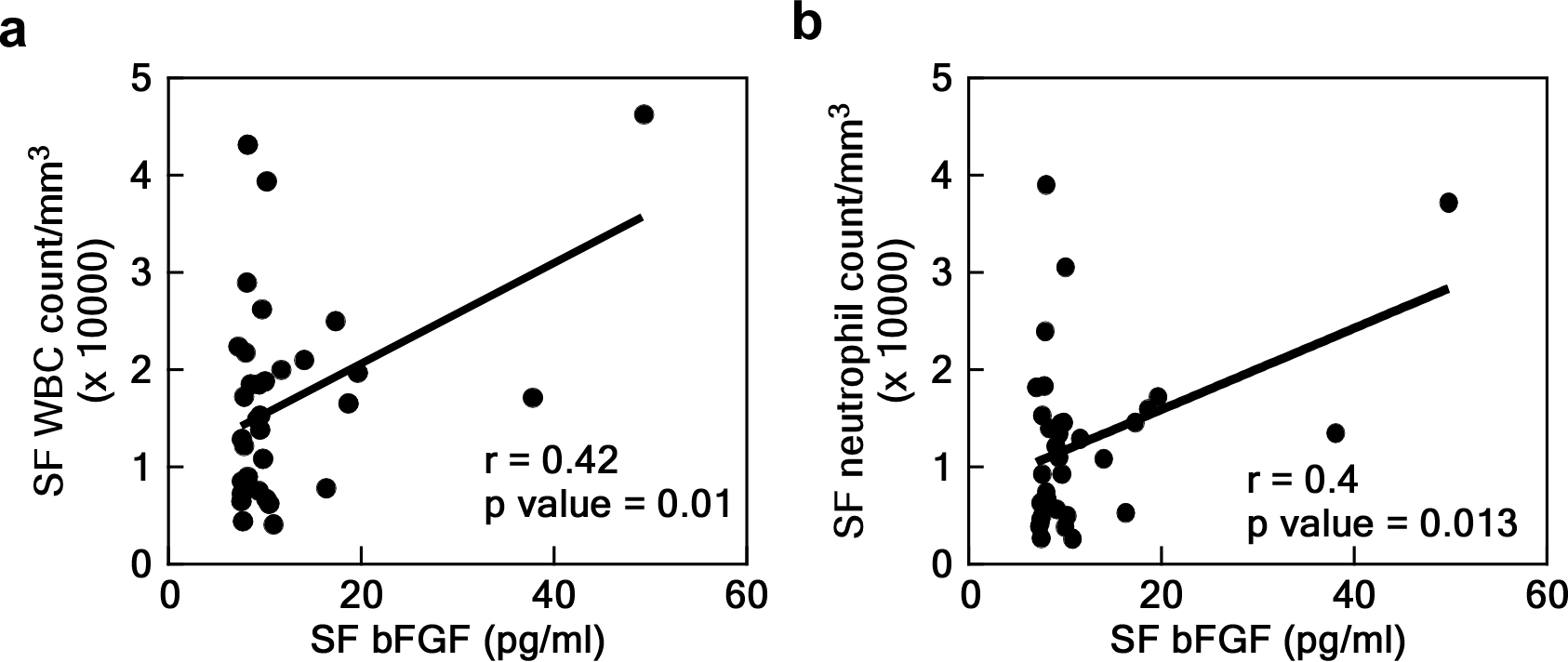


**Supplementary Figure S1.** Correlation between bFGF concentration in RA synovial fluids (SFs) and SF white blood cell counts (**a**) or neutrophil counts (**b**). r = Spearman’s correlation coefficient, n = 36.


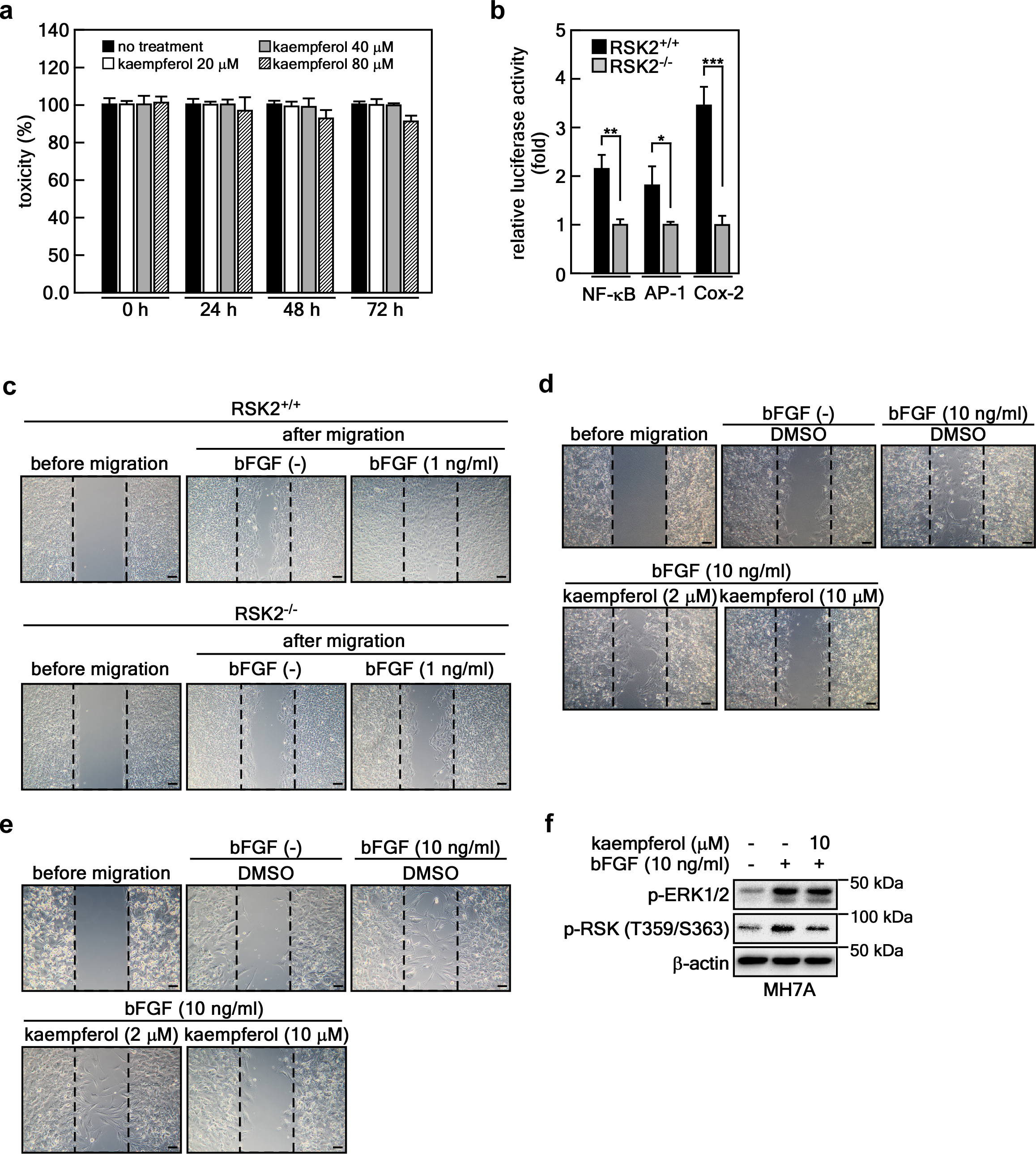


**Supplementary Figure S2** RSK2 mediates bFGF-stimulated cell migration. (**a**) Cytotoxicity of kaempferol in FLSs. FLS cells (3 × 10^4^/cm^2^) were seeded in 96-well plates and cultured overnight. The cells were then treated with the indicated doses of kaempferol, and toxicity was measured at 24, 48, and 72 h by MTS assay. Data were obtained from three independent experiments, and there was no biological significance by the Student’s *t*-test. (**b**) RSK2 mediates transactivation activities of NF-κB and AP-1 and *Cox-2 promoter* activity. Each *pNF-kB-luc*, *pAP-1-luc*, and *pCox-2-p-luc* vector was transfected into RSK2^+/+^ and RSK2*^-/-^* MEFs with pRenilla-luc (internal control for equal transfection), respectively. Firefly luciferase activity was measured at 24 h after transfection in naïve cell culture conditions and normalized Renilla luciferase activity. Data were obtained from three independent experiments, and values are represented as means ± SEM. **P*<0.05; ***P*<0.01; ****P*<0.001 by Student’s *t*-test. (**c**) Representative photographs of the effects of bFGF-induced cell migration in RSK2^+/+^ and RSK2^-/-^ MEFs. The migrated area was quantified by measuring the uncovered area of the wound using Image J (Ver. 1.6), and is presented in Figure 4c. Scale bars, 100 μm. (**d–e**) Efficacy of kaempferol on the cell migration of MH7A (**d**) and human FLSs (**e**). The migrated area was quantified by measuring the uncovered area of the wound using Image J (Ver. 1.6), and is presented in Figure 4d. Scale bars, 100 μm. (**f**) The inhibitory effects of kaempferol on bFGF-induced ERK1/2 and RSK phosphorylation was analyzed by western blotting in MH7A. Data are representative blots obtained from three independent experiments.

**
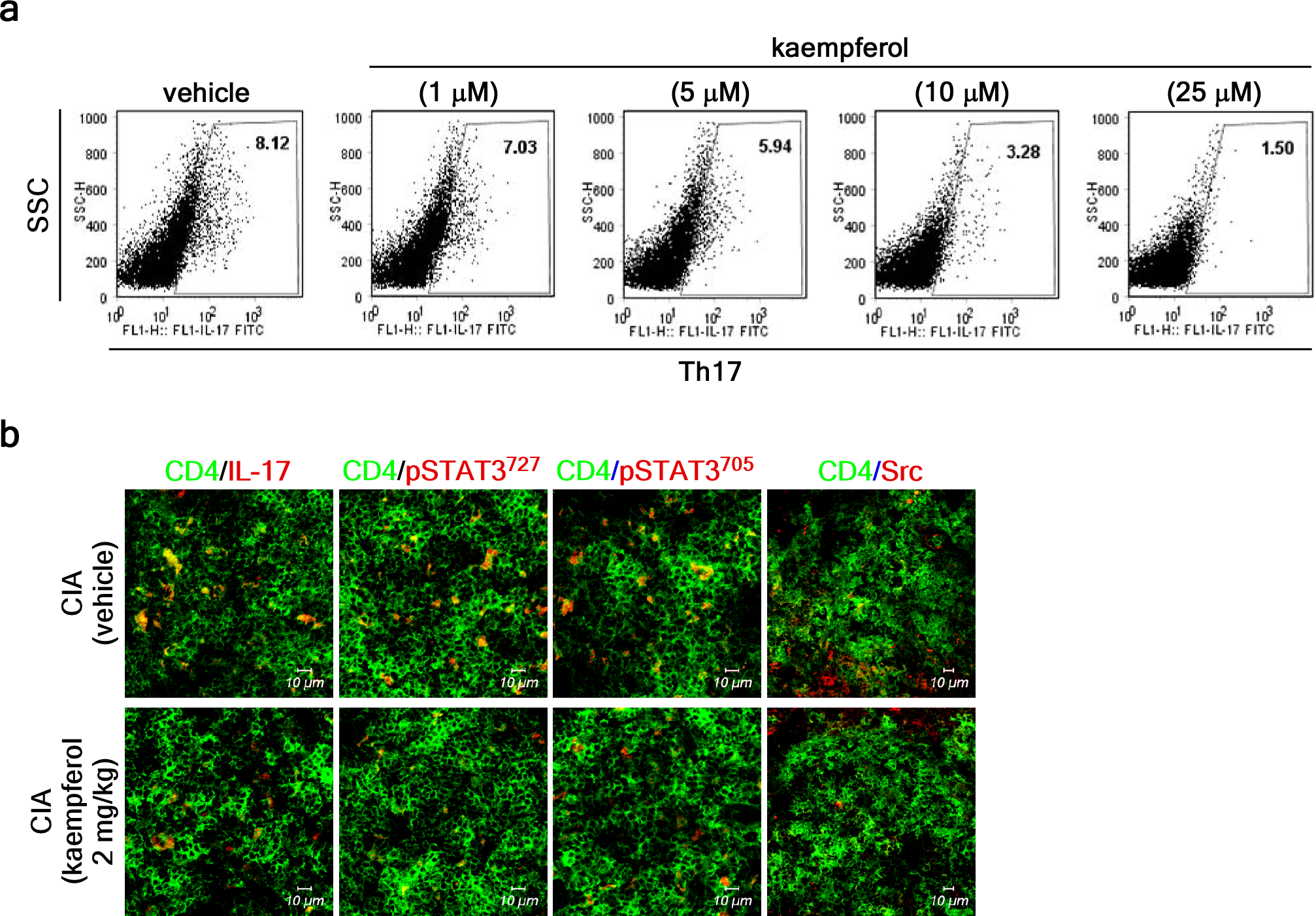
**

**Supplementary Figure S3** Kaempferol inhibits Th17 cell differentiation *in vitro* and *in vivo*. (**a**) Flow cytometry analysis of the inhibitory effects of kaempferol on the Th17 cell-polarizing condition. Data are representative diagrams obtained from three independent experiments, and the values are represented in Figure 5a. (**b**) Representative immunohistofluorescence confocal images of spleen tissues obtained from CIA and CIA+kaempferol mice. Photographs were obtained from three random areas of each specimen. Green color, CD4 staining; red color, indicated target proteins, such as IL-17, pSTAT3^727^, pSTAT3^705^, and Src, respectively. Scale bars, 10 μm. Values are represented in Figure 5f.

**2. Supplementary Western Blot Data**

**[Supplementary Original Western Blot Data]**


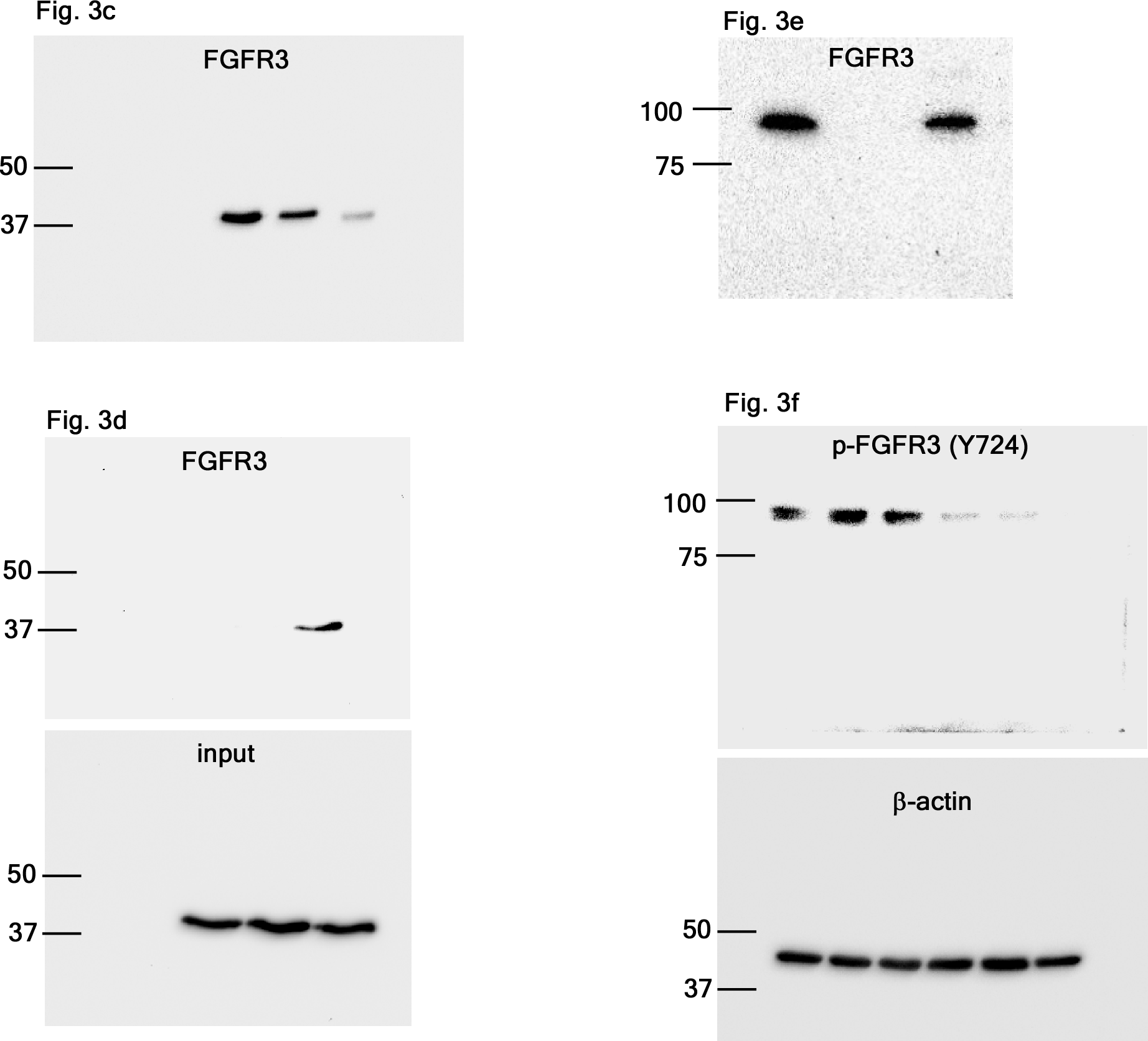


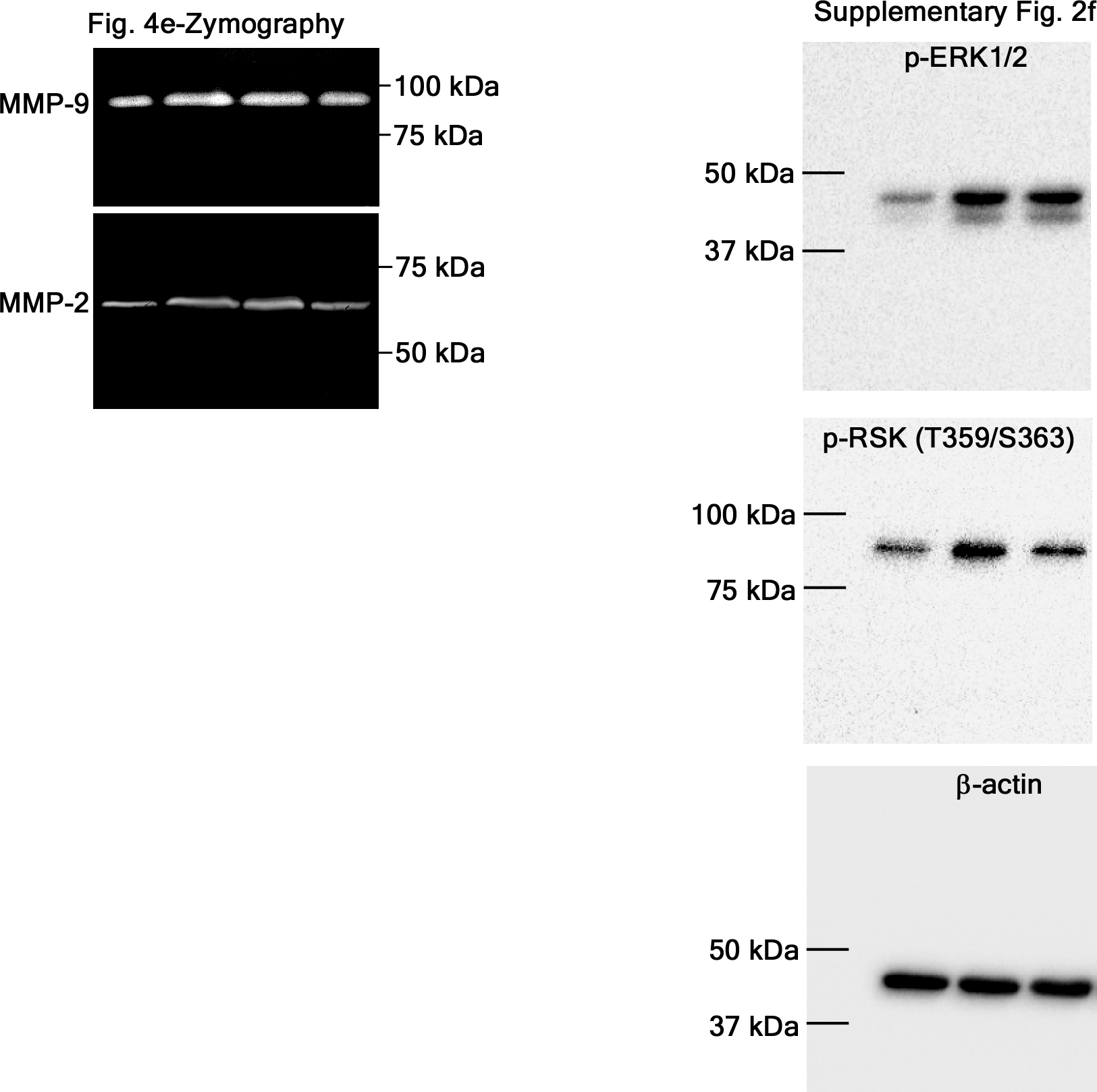

Supplement: Supplementary file 1 — Supplementary Information(DOC 5497 kb) [file 41419_2018_433_MOESM1_ESM.doc]
